# *StrokeClassifier:* ischemic stroke etiology classification by ensemble consensus modeling using electronic health records

**DOI:** 10.1038/s41746-024-01120-w

**Published:** 2024-05-17

**Authors:** Ho-Joon Lee, Lee H. Schwamm, Lauren H. Sansing, Hooman Kamel, Adam de Havenon, Ashby C. Turner, Kevin N. Sheth, Smita Krishnaswamy, Cynthia Brandt, Hongyu Zhao, Harlan Krumholz, Richa Sharma

**Affiliations:** 1 Department of Genetics and Yale Center for Genome Analysis, Yale School of Medicine, New Haven, CT USA; 2 Department of Neurology and Comprehensive Stroke Center, Massachusetts General Hospital and Harvard Medical School Boston, Boston, MA USA; 3 Department of Neurology, Yale School of Medicine, New Haven, CT USA; 4 Department of Neurology, Weill Cornell Medicine, New York City, NY USA; 5 Departments of Genetics and Computer Science, Yale School of Medicine, New Haven, CT USA; 6 Department of Biomedical Informatics and Data Science, Yale School of Medicine, New Haven, CT USA; 7 Departments of Biostatistics, Yale School of Public Health, New Haven, CT USA; 8 Department of Internal Medicine, Yale School of Medicine, New Haven, CT USA

**Keywords:** Preventive medicine, Stroke

## Abstract

Determining acute ischemic stroke (AIS) etiology is fundamental to secondary stroke prevention efforts but can be diagnostically challenging. We trained and validated an automated classification tool, *StrokeClassifier*, using electronic health record (EHR) text from 2039 non-cryptogenic AIS patients at 2 academic hospitals to predict the 4-level outcome of stroke etiology adjudicated by agreement of at least 2 board-certified vascular neurologists’ review of the EHR. *StrokeClassifier* is an ensemble consensus meta-model of 9 machine learning classifiers applied to features extracted from discharge summary texts by natural language processing. *StrokeClassifier* was externally validated in 406 discharge summaries from the MIMIC-III dataset reviewed by a vascular neurologist to ascertain stroke etiology. Compared with vascular neurologists’ diagnoses, *StrokeClassifier* achieved the mean cross-validated accuracy of 0.74 and weighted F1 of 0.74 for multi-class classification. In MIMIC-III, its accuracy and weighted F1 were 0.70 and 0.71, respectively. In binary classification, the two metrics ranged from 0.77 to 0.96. The top 5 features contributing to stroke etiology prediction were atrial fibrillation, age, middle cerebral artery occlusion, internal carotid artery occlusion, and frontal stroke location. We designed a certainty heuristic to grade the confidence of *StrokeClassifier’s* diagnosis as non-cryptogenic by the degree of consensus among the 9 classifiers and applied it to 788 cryptogenic patients, reducing cryptogenic diagnoses from 25.2% to 7.2%. *StrokeClassifier* is a validated artificial intelligence tool that rivals the performance of vascular neurologists in classifying ischemic stroke etiology. With further training, *StrokeClassifier* may have downstream applications including its use as a clinical decision support system.

## Introduction

Identifying the etiology of an ischemic stroke is a clinically challenging and consequential task. In the United States, there are nearly 676,000 cases of ischemic stroke per year^1^, a quarter of whom have had a prior stroke^2^. Among stroke survivors, another stroke can lead to death or further disability. The causative mechanism or etiology of an ischemic stroke can be heterogeneous, including large artery atherosclerosis, cardioembolism, small vessel disease, and other rare, determined etiologies^3^. Nearly 20–30% of ischemic stroke patients in the U.S. are considered cryptogenic with no etiology determined after evaluation^4–11^. The risk of recurrent stroke after a cryptogenic stroke is heightened at 5.6% at 3 months and between 14 and 20% at 2 years^12,13^. In one study, at 21 months, cryptogenic strokes were associated with a higher risk of recurrent stroke in comparison with cardioembolic (HR 1.83, *p* = 0.028) and non-cardioembolic stroke patients with known source (HR 2.4, *p* = 0.046). An analysis of the NOR-FIB study demonstrated an annual risk of stroke recurrence of 7.7% versus 2.8% among individuals with cryptogenic versus non-cryptogenic strokes, respectively^14^. In the Athens Stroke Registry, the stroke recurrence rate in patients with cryptogenic stroke was 29% over a mean of 30.5 months, significantly higher compared with all non-cardioembolic stroke subtypes^15^.

The diagnosis of ischemic stroke etiology determined by a patient’s treating clinician may partly contribute to the differential rates of stroke recurrence by etiology, as each diagnosis prompts a specific secondary stroke prevention treatment plan. Evidence-based, etiology-specific treatments that are proven to reduce the risk of recurrent stroke to varying degrees include carotid revascularization for symptomatic severe carotid stenosis, anticoagulation for atrial fibrillation or left ventricular thrombus, dual antiplatelet therapy after intracranial stenosis-related stroke, and patent foramen ovale closure when it is implicated, among others^16^ (Supplementary Notes). Despite high-level evidence supporting the efficacy of such therapies to prevent recurrent stroke, secondary stroke prevention treatments are significantly underutilized both in the U.S. and globally after an ischemic stroke^17–20^. This implementation gap may underlie the observation that the majority of recurrent strokes are from the same etiology as the index stroke^21^. Furthermore, a cryptogenic stroke diagnosis precludes the institution of any guideline-recommended therapy that targets specific stroke mechanisms and reduces the risk of recurrent stroke from culprit sources^16^. The ability to tailor and implement secondary stroke prevention strategies fundamentally hinges on the diagnosis of the culprit mechanism of an ischemic stroke.

To determine the causative mechanism of an ischemic stroke, clinicians synthesize a vast array of data, including clinical history and physical examination, laboratory data, cardiac rhythm interrogation, cardiac imaging, and neuroradiologic studies. Utilization of diagnostic tools has increased with time. Nevertheless, a significant proportion of patients remain cryptogenic^22^. Diagnostic uncertainty arises due to (1) an inadequate or incomplete workup with further results pending after discharge, (2) a complete workup yielding no known stroke etiology, or (3) multiple, competing possible etiologies, resulting in a diagnosis of stroke of undetermined etiology^3^. An exacerbating factor may be the lack of widespread neurovascular experts specifically trained to collect and examine data to ascertain stroke etiology. A study has demonstrated that compared to evaluation by a non-vascular neurologist, evaluation by a vascular neurologist was associated with a more comprehensive diagnostic investigation that may change management^23^. There is a shortage of vascular neurologists in the United States, with only one in every 6 ischemic stroke patients treated by a board-certified vascular neurologist^23^. In this context, there is an opportunity for an automated, artificial intelligence solution to standardize the process of diagnosing the causative mechanism of stroke.

Artificial intelligence has been heavily adapted for clinical use to help determine patient eligibility for acute stroke therapies such as thrombectomy to abort a stroke, but only minimally for the purpose of stroke prevention^24–26^. There have been several studies of machine learning classifiers to predict stroke etiology. However, these have been limited by the use of manually curated discrete features, single-center samples, insufficient adjudication of stroke etiology outcomes, exclusion of patients with multiple potential etiologies, reliance on a singular model, lack of model explainability, or broad, heterogeneous categorization of stroke etiology^27–35^. In this multi-center study, we aim to develop and externally validate a multi-level, automated ischemic stroke etiology classifier by applying natural language and innovative machine learning tools applied directly to semi-structured text data from the EHR compiled during the AIS hospitalization.

## Results

### Study participants

**Fig. 1:**
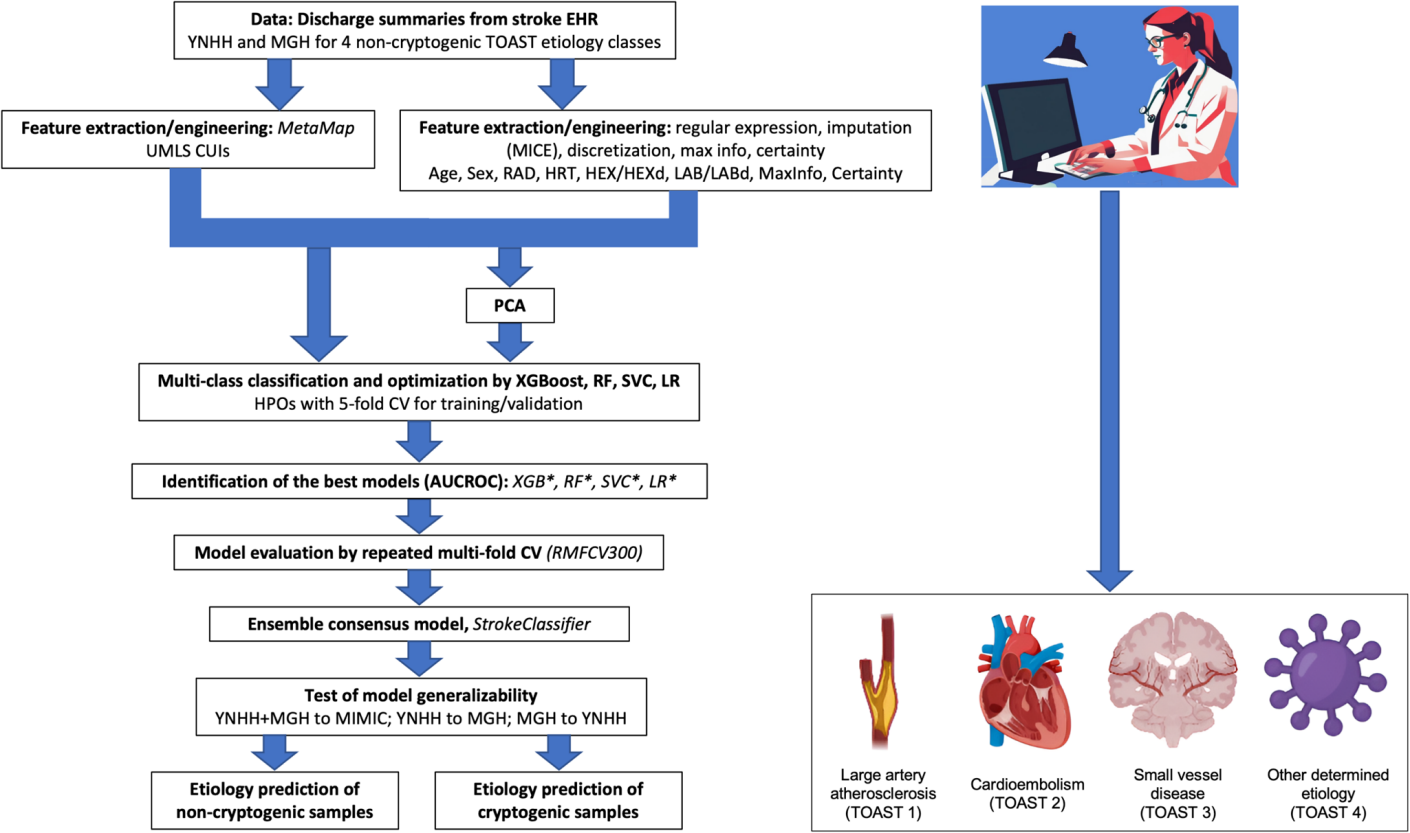
Workflow overview. Icons were created with BioRender.com.

The study sample consisted of 3,262 discharge summaries with AIS diagnoses (*N* = 1269 at YNHH from 2015 to 2020; *N* = 1493 at MGH from 2016 to 2019; *N* = 500 at BIDMC from 2001 to 2012). The characteristics of the three cohorts are presented in Table 1. The derivation cohorts of YNHH and MGH as input for model development (Fig. 1) were similar, with some exceptions. The YNHH cohort was significantly older (median age 71 years [IQR 59–82]) compared with the MGH cohort (median age 69 [IQR 59–79]) (*p* = 0.013). The median word count of the YNHH discharge summaries (1639 words [IQR 1274–2064]) was significantly lower than in the MGH discharge summaries (2058 words [IQR 1593–2554]) (*p* = 1.21e−35). The YNHH cohort was significantly more likely than the MGH cohort to have hyperlipidemia (32.9% versus 11.5%, *p* = 0.001) and coronary artery disease (17.8% versus 4.0%, *p* = 0.003). The YNHH and MGH cohorts had similar distributions of stroke etiologies adjudicated by vascular neurologists: large artery atherosclerosis (19.8% versus 21.0%), cardioembolism (32.9% versus 29.9%), small vessel disease (15.3% versus 10.7%), other determined etiology (8.9% versus 9.6%), and cryptogenic etiology (23.1% versus 28.8%). The degree of completeness of extracted features was comparable between YNHH and MGH with respect to UMLS CUIs (extracted from 95.7% versus 94.5%), neuroimaging features (extracted from 94.1% versus 92.0%), cardiac features (95.4% versus 93.0%), clinical history (90.3% versus 91.5%), and laboratory features (90.0% versus 92.3%).

**Table 1:** Description of study cohorts

	Data for model development			Data for external validation	*P*-value	
	YNHH (*N* = 1269)	MGH (*N* = 1493)	YNHH + MGH (*N* = 2762)	MIMIC from BIDMC (*N* = 500)	YNHH vs. MGH	YNHH + MGH vs. MIMIC
Age (median [IQR Q1-Q3])	71 [59–82]	69 [59–79]	70 [59–80]	73 [61–82]	**0.01295**	0.45335
Male sex	636 (50.1%)	812 (54.4%)	1448 (52.4%)	232 (46.4%)	1	1
Race (White; Black or African American; Others)	891 (70.2%); 273 (21.5%); 105 (8.3%)	1095 (73.3%); 107 (7.2%); 291 (19.5%)	1986 (71.9%); 380 (13.8%); 396 (14.3%)	NA	0.94280	NA
Admission Year	2015–2020	2016–2019	2015–2020	2001–2012	NA	NA
Characters in discharge summary texts (median [IQR Q1-Q3])	11294 [8865–14033]	13338 [10366–16508]	12255 [9530–15590]	11436 [7650–15184]	**2.57E−18**	**0.00457**
Words in discharge summary texts (median [IQR Q1-Q3])	1639 [1274–2064]	2058 [1593–2554]	1846 [1410–2365]	1712 [1160–2294]	**1.21E−35**	**0.00214**
NIHSS (median [IQR Q1-Q3]; %N)	5 [1–11]; 68.7%	6 [2–15]; 34.3%	5 [2–13]; 50.1%	16 [10–20]; 9.2%	NA	NA
*Co-morbidities (CUI freq.)*					**0.00721**	0.31430
Hypertension	1006 (82.1%)	1118 (78.5%)	2124 (80.2%)	384 (77.0%)	0.77635	0.79867
Hyperlipidemia	403 (32.9%)	164 (11.5%)	567 (21.4%)	98 (19.6%)	**0.00132**	0.78388
Diabetes	571 (46.6%)	505 (35.4%)	1076 (40.6%)	214 (42.9%)	0.21615	0.80278
Atrial fibrillation	476 (38.9%)	739 (51.9%)	1215 (45.8%)	215 (43.1%)	0.17248	0.76954
Cigarette use	1 (0.1%)	0 (0%)	1 (0.04%)	0 (0%)	0.75183	0.84597
Drug use	14 (1.1%)	32 (2.2%)	46 (1.7%)	10 (2.0%)	0.54483	0.88972
Coronary artery disease	218 (17.8%)	57 (4.0%)	275 (10.4%)	104 (20.8%)	**0.00312**	0.06109
Heart failure	174 (14.2%)	157 (11.0%)	331 (12.5%)	136 (27.3%)	0.52383	**0.01919**
*Stroke etiology*					0.80070	**0.00103**
Large artery atherosclerosis (1)	251 (19.8%)	314 (21.0%)	565 (20.5%)	44 (8.8%)	0.84461	**0.03066**
Cardioembolism (2)	418 (32.9%)	446 (29.9%)	864 (31.3%)	256 (51.2%)	0.69881	**0.02846**
Small vessel disease (3)	194 (15.3%)	160 (10.7%)	354 (12.8%)	18 (3.6%)	0.37006	**0.02310**
Other determined (4)	113 (8.9%)	143 (9.6%)	256 (9.3%)	88 (17.6%)	0.87554	0.10953
Cryptogenic (5)	293 (23.1%)	430 (28.8%)	723 (26.2%)	94 (18.8%)	0.42780	0.26997
*Degree of feature completene*ss					0.99900	0.97650
UMLS CUIs (CUI)	1215 (95.7%)	1425 (94.5%)	2626 (95.1%)	499 (99.8%)	0.92854	0.73506
Neuroimaging (RAD)	1194 (94.1%)	1373 (92.0%)	2567 (92.9%)	484 (96.8%)	0.87606	0.77930
Cardiac data (HRT)	1210 (95.4%)	1389 (93.0%)	2599 (94.1%)	492 (98.4%)	0.86597	0.75654
Clinical History (HEX)	1146 (90.3%)	1366 (91.5%)	2512 (90.9%)	453 (90.6%)	0.92989	0.97936
Laboratory data (LAB)	1142 (90.0%)	1378 (92.3%)	2520 (91.2%)	425 (85.0%)	0.86443	0.63842
*MetaMap*						
Processing time on average (min)	5.0	3.3	4.1	0.8		

N.B. chi-squared tests for categorical variables and Student’s *t*-tests for numerical variables. Those *p*-values < 0.05 are highlighted in bold.

**Fig. 2:**
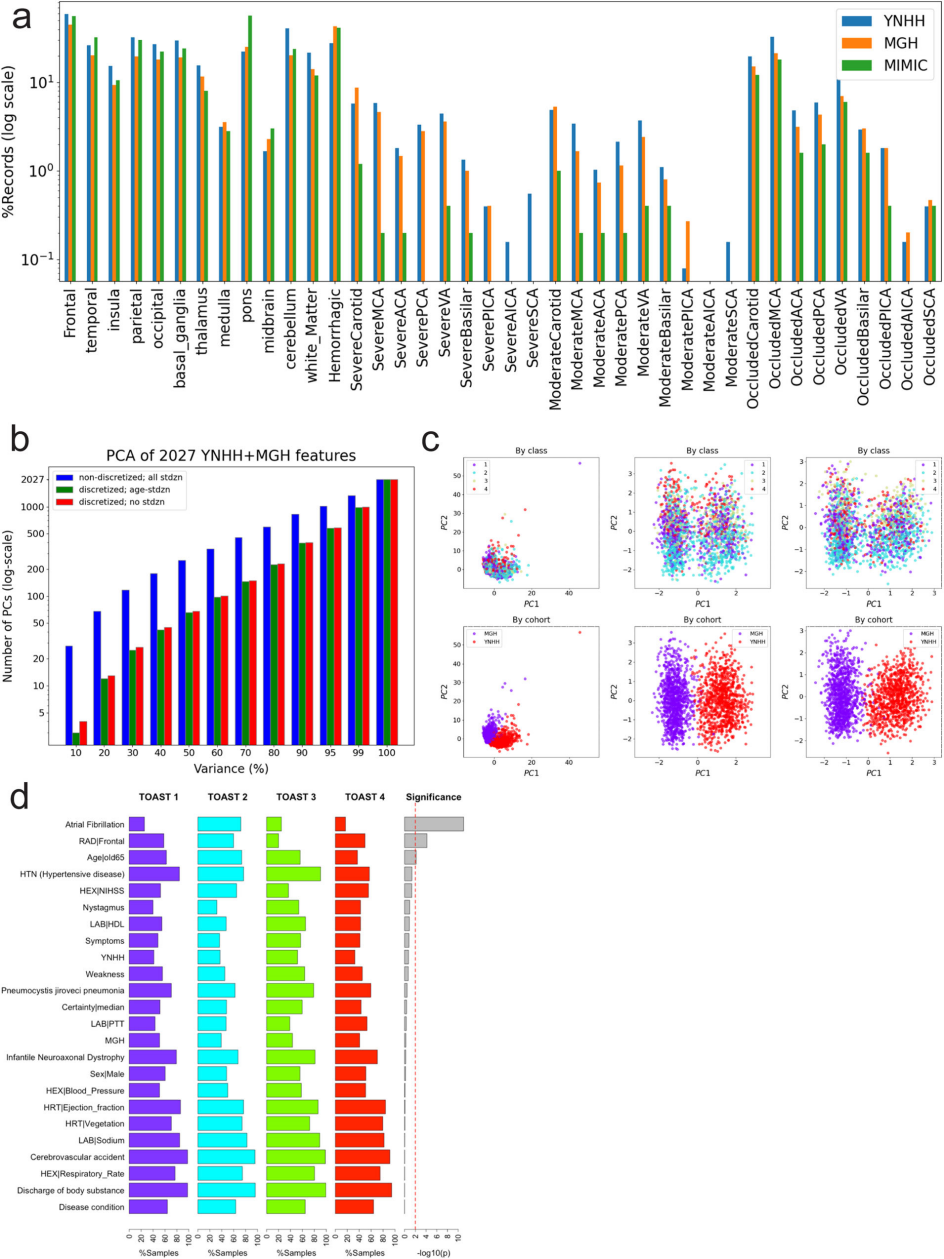
Exploratory data analysis. **a** Percentage comparison of discharge summary records with radiology-related features among the three cohorts. **b** Numbers of PCs for each PCA total variance cutoff for 2027 YNHH and MGH features in the case of non-discretized features with all standardized continuous features, discretized features with the standardized age feature, and discretized features with no standardization. **c** Scatter plots of PC1 and PC2 for the three cases in **b** by class and by cohort. **d** Top features that are present in >50% of non-cryptogenic stroke records for each TOAST class and their significance by chi-squared tests.

Characteristics of the combined derivation cohort were compared with those of the external validation MIMIC-III cohort. The external validation cohort was comparable in age to the combined derivation cohort. The median word count of the external validation cohort discharge summaries was significantly lower (1712 words [IQR 1160–2294], *p* = 0.002). The external validation cohort was more likely to have heart failure (27.3% versus 12.5%, *p* = 0.019). The distribution of stroke etiologies differed significantly between the derivation and external validation cohorts (*p* = 0.001). Large artery atherosclerosis (8.8% versus 20.5%, *p* = 0.031) and small vessel disease (3.6% versus 12.8%, *p* = 0.023) were significantly less frequent in the external validation cohort, while cardioembolism was significantly more frequent (51.2% versus 31.3%, *p* = 0.028). The derivation and external validation cohorts were similar in terms of feature completeness (*p* = 0.638–0.979) (Table 1; Fig. 2a).

### Data post-processing and principal component analysis

Of the 2039 non-cryptogenic stroke samples in the YNHH and MGH cohorts, 1932 samples were successfully post-processed by MetaMap (see “Methods”) as input for model development (Fig. 1). Imputation of missing entries in categorical and numerical features was performed using MICE in the derivation cohort of 1932 samples and Random Forests-based imputation in the external validation cohort (see “Methods”; Supplementary Table 3). The levels of missingness for the categorical and numerical features were 91.9% (76.8% to 99.9%) and 73.4% (2.3% to 99.9%) on average, respectively. Imputation of several features failed, and they were excluded subsequently. All subsequent analyses were performed on the imputed datasets.

For the derivation cohort analyzed for model development, we performed PCA on all of the 2027 features, either discretized or not, to reduce dimensionality or noise. We then selected the top PCs for each of the ten thresholds of the total variance (see “Methods”) for alternative model development (Fig. 1). We found that 99% of the total variance could be explained by less than half of all features, the first principal component with about 4.5% variance discriminating between the two cohorts (Fig. 2b, c).

### Base models with optimized hyperparameters and model performances

**Fig. 3:**
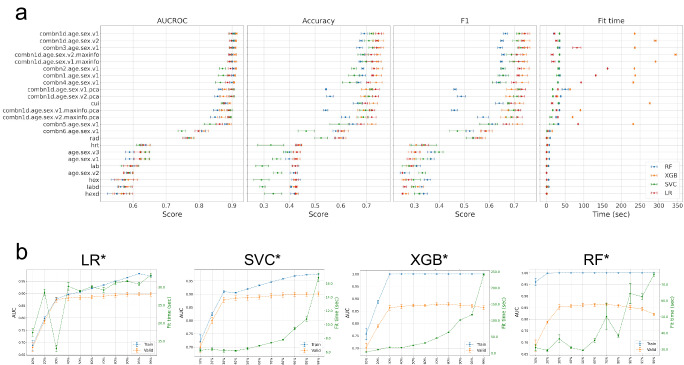
Model performances. **a** Performances and fit times of each optimized model for each feature group by fivefold CV. **b** AUCROC and fit times of the PCA-based optimized models with combn1d.age.sex.v1 (${{\boldsymbol{\Lambda }}}_{{\boldsymbol{1}}}$). The error bars represent the mean ± standard deviation (SD) of the fivefold CV.

We performed 96 hyperparameter optimizations (HPOs) for the 4 supervised machine-learning algorithms of LR, SVC, RF, and XGB and 24 training datasets (Table 2A and Supplementary Table 4; Figs. 1 and 3a). Based on the AUCROC rankings in the fivefold CV (Supplementary Table 5), we denote the best model for each of the four strategies as LR*, SVC*, RF*, and XGB*, respectively, hereafter. All four best models were built using the full features with discretization (age + sex + CUI + RAD + HRT + HEXd + LABd, denoted by *combn1d.age.sex.v1* or **Λ**_**1**_) (Table 2A). AUCROC and mean cross-validated accuracy were 89.8% and 74.7% for LR*, 90.1% and 71.9% for SVC*, 91.3% and 74.6% for XGB*, and 90.5% and 69.1% for RF*. Similar performances were observed with PCA of the full features (denoted by **Λ**_**1_pca**_), except for RF* (Table 2A). Fit times for XGB* with **Λ**_**1**_ were particularly longer (>235 s) than those for the other three models (Table 2A). We also observe that XGB and RF tend to overfit (Fig. 3b and Supplementary Fig. 2). CUIs contributed most to model performance as measured by AUCROC, while the radiologic features ranked second. The decrease in performance was the largest for each model when CUIs were excluded from the full feature group. On the other hand, excluding the LAB and HEX features tend to improve the performance. There was no performance improvement with those samples of high-feature information defined by the presence of at least four feature groups.

**Table 2:** Optimized model performances

(A) Validation results of the optimized base models for each feature group																	
Feature group ID	Feature group elements	Fit time (s)				AUCROC				Accuracy				F1			
		LR*	SVC*	XGB*	RF*	LR*	SVC*	XGB*	RF*	LR*	SVC*	XGB*	RF*	LR*	SVC*	XGB*	RF*
age.sex.v1	Age + Sex	0.314	0.717	1.358	7.755	0.620	0.637	0.636	0.589	0.425	0.350	0.427	0.398	0.283	0.335	0.300	0.366
age.sex.v2	Age (binarized) + Sex	0.111	0.370	0.408	0.761	0.585	0.589	0.586	0.583	0.424	0.353	0.424	0.417	0.252	0.332	0.252	0.262
age.sex.v3	Age (standardized) + Sex	0.109	0.773	1.341	7.471	0.622	0.637	0.636	0.589	0.429	0.391	0.427	0.399	0.305	0.399	0.300	0.367
hex	HEX	0.720	0.869	1.051	6.314	0.573	0.576	0.585	0.568	0.425	0.290	0.421	0.427	0.276	0.297	0.257	0.353
hexd	HEXd	0.114	0.829	1.786	0.954	0.571	0.570	0.565	0.550	0.423	0.337	0.427	0.420	0.252	0.319	0.266	0.337
lab	LAB	0.226	1.338	2.033	6.001	0.593	0.591	0.596	0.599	0.422	0.294	0.422	0.423	0.287	0.279	0.290	0.308
labd	LABd	0.122	1.464	3.586	6.693	0.578	0.575	0.584	0.562	0.426	0.295	0.417	0.407	0.262	0.297	0.314	0.330
hrt	HRT	2.148	1.369	3.486	7.686	0.631	0.619	0.631	0.638	0.435	0.327	0.439	0.435	0.343	0.338	0.307	0.308
rad	RAD	0.110	1.030	3.709	9.723	0.764	0.769	0.775	0.771	0.593	0.523	0.600	0.593	0.554	0.535	0.566	0.554
cui	CUIs	15.291	32.001	275.934	34.236	0.876	0.872	0.892	0.880	0.695	0.674	0.727	0.665	0.688	0.677	0.722	0.640
combn1.age.sex.v1	Age + Sex + HEX + LAB + HRT + RAD + CUIs	131.999	32.179	236.997	35.784	0.891	0.873	0.908	0.904	0.724	0.652	0.739	0.684	0.716	0.656	0.733	0.655
combn2.age.sex.v1	Age + Sex + HEX + HRT + RAD + CUIs	163.475	32.328	234.616	34.418	0.895	0.871	0.911	***0.905***	0.730	0.651	0.745	0.685	0.722	0.654	0.739	0.657
combn3.age.sex.v1	Age + Sex + LAB + HRT + RAD + CUIs	81.605	30.247	235.918	35.326	0.896	0.898	0.910	***0.905***	0.740	***0.725***	***0.748***	0.673	0.735	***0.727***	***0.743***	0.644
combn4.age.sex.v1	Age + Sex + HEX + LAB + RAD + CUIs	92.858	30.702	232.069	31.381	0.887	0.865	0.906	0.900	0.706	0.635	0.737	0.669	0.699	0.640	0.732	0.637
combn5.age.sex.v1	Age + Sex + HEX + LAB + HRT + CUIs	84.851	19.786	231.799	32.263	0.862	0.833	0.892	0.882	0.678	0.593	0.720	0.647	0.669	0.598	0.713	0.612
combn6.age.sex.v1	Age + Sex + HEX + LAB + HRT + RAD	4.455	1.621	15.531	9.306	0.798	0.747	0.815	0.808	0.602	0.465	0.608	0.580	0.584	0.473	0.589	0.522
***combn1d.age.sex.v1***	Age + Sex + HEXd + LABd + HRT + RAD + CUIs	***21.406***	***35.117***	235.597	***19.809***	***0.898***	***0.901***	***0.913***	***0.905***	***0.747***	0.719	0.746	***0.691***	***0.744***	0.721	0.741	***0.665***
combn1d.age.sex.v2	Age (binarized) + Sex + HEXd + LABd + HRT + RAD + CUIs	26.649	35.415	290.860	34.993	***0.898***	0.900	0.912	0.904	0.741	0.709	0.746	0.679	0.735	0.712	0.741	0.651
combn1d.age.sex.v1.maxinfo	Age + Sex + HEXd + LABd + HRT + RAD + CUIs with MaxInfo	18.608	34.546	291.177	33.070	0.893	0.894	0.908	0.901	0.723	0.698	0.740	0.677	0.718	0.700	0.735	0.649
combn1d.age.sex.v2.maxinfo	Age (binarized) + Sex + HEXd + LABd + HRT + RAD + CUIs with MaxInfo	15.444	35.102	344.686	35.421	0.893	0.894	0.908	0.901	0.718	0.686	0.743	0.685	0.712	0.689	0.738	0.657
combn1d.age.sex.v1.pca	Age (standardized) + Sex + HEXd + LABd + HRT + RAD + CUIs with PCA	30.833	16.843	63.394	50.176	0.897	0.900	0.878	0.864	0.735	0.718	0.698	0.543	0.731	0.720	0.689	0.463
combn1d.age.sex.v2.pca	Age (binarized) + Sex + HEXd + LABd + HRT + RAD + CUIs with PCA	26.028	17.572	29.503	38.544	0.897	0.899	0.873	0.856	0.735	0.710	0.691	0.558	0.730	0.712	0.682	0.488
combn1d.age.sex.v1.maxinfo.pca	Age (standardized) + Sex + HEXd + LABd + HRT + RAD + CUIs with MaxInfo and PCA	27.267	17.190	69.948	34.900	0.890	0.893	0.868	0.856	0.722	0.696	0.679	0.541	0.717	0.699	0.666	0.460
combn1d.age.sex.v2.maxinfo.pca	Age (binarized) + Sex + HEXd + LABd + HRT + RAD + CUIs with MaxInfo and PCA	30.783	12.510	90.964	25.857	0.891	0.894	0.869	0.848	0.721	0.687	0.689	0.618	0.714	0.690	0.676	0.576

(B) Validation results of the ensemble/meta models using combn1d.age.sex.v1 (Λ_1_)							
Model	AUCROC	AUPRC	ACC	BA	PRC	F1	KAPP
LR*	0.898 ± 0.008	0.796 ± 0.012	0.747 ± 0.012	0.706 ± 0.021	0.747 ± 0.011	0.744 ± 0.013	0.632 ± 0.019
SVC*	0.900 ± 0.009	0.802 ± 0.014	0.728 ± 0.007	0.693 ± 0.011	0.726 ± 0.009	0.725 ± 0.008	0.607 ± 0.011
SVC2	0.887 ± 0.009	0.772 ± 0.014	0.719 ± 0.010	0.726 ± 0.007	0.733 ± 0.011	0.721 ± 0.011	0.606 ± 0.014
XGB*	0.913 ± 0.003	0.827 ± 0.014	0.746 ± 0.023	0.697 ± 0.037	0.745 ± 0.026	0.741 ± 0.025	0.627 ± 0.035
RF*	0.905 ± 0.005	0.817 ± 0.010	0.691 ± 0.013	0.582 ± 0.012	0.738 ± 0.016	0.665 ± 0.010	0.523 ± 0.021
**MAX**	0.907 ± 0.006	0.821 ± 0.012	0.736 ± 0.009	**0.729** ± **0.005**	0.740 ± 0.010	0.736 ± 0.010	0.626 ± 0.012
**MIN**	0.907 ± 0.007	0.818 ± 0.011	0.743 ± 0.009	0.709 ± 0.015	0.743 ± 0.011	0.740 ± 0.010	0.628 ± 0.014
**MEAN**	**0.912** ± **0.005**	**0.826** ± **0.009**	**0.750** ± **0.008**	0.725 ± 0.008	**0.749** ± **0.009**	**0.748** ± **0.009**	**0.640** ± **0.011**
**MEDIAN**	0.910 ± 0.005	0.823 ± 0.008	0.749 ± 0.009	0.714 ± 0.012	0.748 ± 0.011	0.746 ± 0.010	0.636 ± 0.014
***StrokeClassifier***	NA	NA	***0.744*** ± ***0.009***	***0.710*** ± ***0.015***	***0.743*** ± ***0.009***	***0.740*** ± ***0.010***	***0.629*** ± ***0.014***

N.B. Those with MaxInfo ≥ 4 are denoted by a suffix of “.maxinfo” in the feature group names. The best performances are highlighted in bold in italics. *LR* logistic regression, *SVC* support vector classifier, *XGB* XGBoost, *RF* Random Forests.

N.B. The values are mean ± standard deviation (SD) for the five validation sets of fivefold CV. The highest mean value for each performance metric is highlighted in bold.

Next, we evaluated the performance of each optimized model for the full cohort of the 1932 samples. We also built and examined the SVC2 model, which calculates alternative prediction probabilities as a different calibration approach using the optimized hyperparameters from SVC* (see “Methods”). The runtimes for the 5 models of LR*, SVC*, RF*, XGB*, and SVC2 were 114 ms, 10.8 s, 258 ms, 475 ms, and 10.8 s, respectively, and their accuracies were 90.4%, 86.2%, 92.4%, 97.6%, and 88.1%. The numbers of samples correctly predicted by *N* = 1, 2, 3, 4, and 5 models (i.e., supports) are 59 (3.1%), 74 (3.8%), 92 (4.8%), 108 (5.6%), and 1574 (81.5%), respectively. In other words, 91.9% of all samples were correctly predicted by at least 3 models. The remaining 25 samples (1.3%) were incorrectly predicted by all the 5 models. The [numbers, percentages] of 1,002 MGH and 930 YNHH samples with *N* = 0 to 5 supports are [(13, 12), (1.3%, 1.3%)], [(32, 27), (3.2%, 2.9%)], [(31, 43), (3.1%, 4.6%)], [(44, 48), (4.4%, 5.2%)], [(57, 51), (5.7%, 5.5%)], and [(825, 749), (82.3%, 80.5%)], respectively. When we analyzed those 59 samples correctly predicted by a single model (*N* = 1), RF* was found to correctly predict 49 (83.1%) samples, in particular for TOAST 1 and 2 (22 and 16 samples or 37.3% and 27.1%, respectively).

### Performance of ensemble models and consensus meta-model, StrokeClassifier

We aggregated the 4 optimized models built using the full features and samples, **Χ(Λ**_**1**_**)**, along with SVC2, into four ensemble models with four pre-specified summary statistics (see “Methods”). The fivefold CV performance metrics associated with these ensemble models are shown in Table 2B. We observed performance improvement using the ensemble models by up to 0.7% on average (F1 score) in MEAN across the 7 metrics compared to the individual base models. No single ensemble model performed better than the rest in predicting each TOAST classification; there was variability among models that predicted each TOAST classification most accurately (Supplementary Tables 5–7). Spearman correlation and Cohen’s kappa values among the 9 base classifiers range from 0.78 and 0.81 (between RF* and SVC2) to 0.96 and 0.97 (between MEAN and MEDIAN), respectively. This observation supported our inclination to utilize a consensus ensemble meta-model, designated as *StrokeClassifier*, to harness the varying predictive capacities of the 9 classifiers while diluting the bias introduced by individual models, bolstering the robustness and generalizability of the model’s output.

*StrokeClassifier* demonstrated the following performance measures on average for predicting the 4-level outcome of non-cryptogenic stroke etiology: accuracy of 0.744, balanced accuracy of 0.710, weighted F1 of 0.740, and Cohen’s kappa of 0.629 (Table 2B), indicating substantial agreement with vascular neurologist-adjudicated stroke etiology. The mean accuracy of *StrokeClassifier* for each specific etiology versus not as a binary outcome ranged from 0.829 for TOAST 2 to 0.913 for TOAST 4 (Table 3).

**Table 3:** Performance of *StrokeClassifier* for each TOAST classification

Physician diagnosis	Accuracy	BA	PPV	F1	Kappa	FPR	FNR
Large artery atherosclerosis (1)	0.836 ± 0.015	0.785 ± 0.033	0.718 ± 0.029	0.834 ± 0.017	0.580 ± 0.049	0.073 ± 0.015	**0.091** ± **0.021**
Cardioembolism (2)	**0.829** ± **0.014**	0.830 ± 0.011	**0.781** ± **0.027**	**0.830** ± **0.013**	0.654 ± 0.025	**0.100** ± **0.018**	0.071 ± 0.007
Small vessel disease (3)	0.909 ± 0.010	**0.854** ± **0.010**	0.733 ± 0.049	0.910 ± 0.008	**0.693** ± **0.024**	0.050 ± 0.015	**0.041** ± **0.006**
Other determined (4)	**0.913** ± **0.006**	**0.764** ± **0.037**	**0.685** ± **0.038**	**0.909** ± **0.008**	**0.568** ± **0.046**	**0.033** ± **0.010**	0.054 ± 0.010

N.B. The values are mean ± SD for five validation sets of fivefold CV. *BA* balanced accuracy = (sensitivity + specificity)/2, *PPV* positive predictive value = precision = 1 − false discovery rate, *Kappa* Cohen’s kappa, *FPR* false positive rate = 1 − true negative rate (or specificity), *FNR* false negative rate = 1 − true positive rate (or sensitivity or recall). The best and worst values for each performance metric are highlighted in bold.

### Performance validation using 300 repeated multi-fold CV splits

**Fig. 4:**
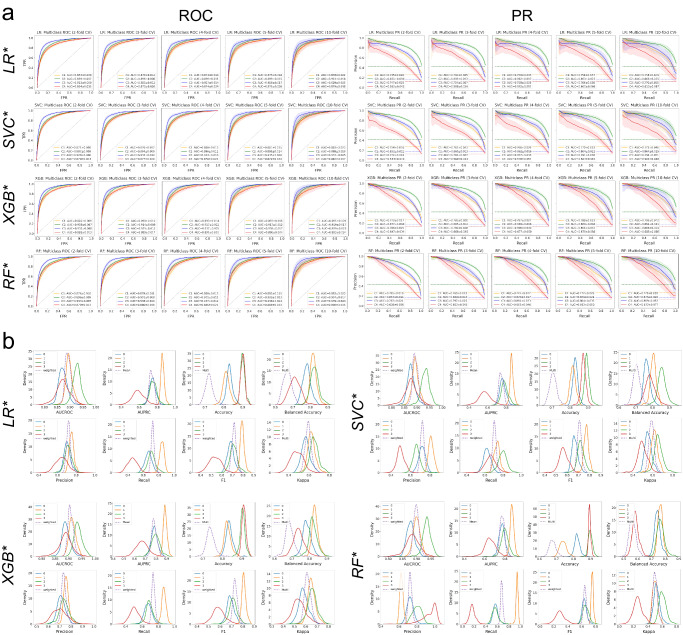
Model validation by RMFCV300. **a** ROC and PR curves for each optimized model and each CV fold by the RMFCV300 strategy. AUCROC and AUPRC are shown for each class vs. the rest. **b** Distributions of multiple performance metrics for each optimized model and each class (vs. the rest) as well as (weighted) averages.

Since cross-validation strategies such as the 5-fold CV used for HPO are anchored to a particular seed number, which is subjective, we used 300 training-validation data splits by repeated multi-fold CV, *RMFCV300*, to derive better estimates of model performance and generalization errors. We performed RMFCV300 for the four best models optimized by the HPO, focusing on model performances by AUCROC and AUPRC metrics (Fig. 4 and Supplementary Fig. 3; Supplementary Tables 8–10). While there was variability in the magnitude of model performance measures for each TOAST class among the four models, all four models performed best in predicting TOAST three in terms of AUCROC, while they performed best in predicting TOAST two in terms of AUPRC, regardless of the number of CV folds employed. For each TOAST class, the means and standard deviations of both AUCROC and AUPRC for the CV fold repetitions consistently increased with the increasing CV folds across the four models.

### Analysis of age-sex-race strata

To evaluate whether there was heterogeneity in model performances based on patient age, sex, and race, we assessed model performances in age-sex-race subgroups using the RMFCV300 validation sets (Table 4 and Supplementary Tables 11–14). We observed that *StrokeClassifier* tended to perform worse in the stratum of males/age ≥ 65, in particular for predicting TOAST 3 and 4 (lowest mean F1 of 64.6% and 36.3% across all strata, respectively). The stratum of Black or African Americans also showed a relatively worse performance for TOAST 1 (lowest mean F1 of 63.8%). In contrast, *StrokeClassifier* performed better in the stratum of females/age < 65, in particular for predicting TOAST 3 and 4 (highest mean F1 of 80.6% and 68.7% across the strata, respectively). We note that all mean performance values were greater than 60%, except F1 scores in TOAST 4 for the 4 strata of male (51.4% ± 8.1%), age ≥ 65 (50.8% ± 10.4%), male/age ≥ 65 (36.3% ± 16.9%), male/age < 65 (56.1% ± 8.9%), white (59.9% ± 6.5%), Black or African American (53.4% ± 21.7%), and others (57.7% ± 13%).

**Table 4:** Performance of *StrokeClassifier* in age-sex-race strata

Performance metric	TOAST	Female	Male	Age ≥ 65	Age < 65	Female, age ≥ 65	Female, age < 65	Male, age ≥ 65	Male, age < 65	White	Black or African American	Others
		*246.8* ± *120.9*	*287.7* ± *140.8*	*330.6* ± *161.7*	*203.9* ± *100.0*	*169.0* ± *83.0*	*77.7* ± *38.5*	*161.6* ± *79.4*	*126.2* ± *62.1*	*385.1* ± *188.2*	*72.5* ± *36.1*	*76.9* ± *38.1*
Accuracy	1	0.862 ± 0.023	0.817 ± 0.019	0.837 ± 0.020	0.839 ± 0.026	0.856 ± 0.027	**0.874** ± **0.040**	0.817 ± 0.030	0.818 ± 0.032	0.840 ± 0.018	**0.817** ± **0.048**	0.845 ± 0.045
	2	0.848 ± 0.023	0.818 ± 0.023	0.834 ± 0.024	0.828 ± 0.027	**0.855** ± **0.029**	0.833 ± 0.040	**0.812** ± **0.036**	0.824 ± 0.033	0.828 ± 0.021	0.837 ± 0.051	0.844 ± 0.045
	3	0.919 ± 0.017	0.894 ± 0.017	0.901 ± 0.016	0.914 ± 0.019	**0.920** ± **0.021**	0.916 ± 0.032	0.880 ± 0.025	0.912 ± 0.028	0.911 ± 0.014	**0.875** ± **0.037**	0.906 ± 0.038
	4	0.918 ± 0.018	0.896 ± 0.018	0.947 ± 0.012	0.839 ± 0.027	**0.956** ± **0.017**	**0.835** ± **0.040**	0.938 ± 0.019	0.842 ± 0.036	0.905 ± 0.016	0.930 ± 0.033	0.887 ± 0.036
Balanced accuracy	1	0.780 ± 0.039	0.782 ± 0.025	0.782 ± 0.026	0.786 ± 0.037	**0.772** ± **0.043**	**0.797** ± **0.069**	0.785 ± 0.036	0.778 ± 0.039	0.788 ± 0.024	0.748 ± 0.065	0.797 ± 0.056
	2	**0.850** ± **0.023**	0.813 ± 0.024	0.835 ± 0.024	0.796 ± 0.034	0.848 ± 0.030	0.810 ± 0.050	0.817 ± 0.035	**0.791** ± **0.044**	0.830 ± 0.021	0.829 ± 0.055	0.845 ± 0.045
	3	0.862 ± 0.034	0.839 ± 0.032	0.820 ± 0.034	0.887 ± 0.029	0.847 ± 0.046	0.883 ± 0.051	**0.794** ± **0.049**	**0.891** ± **0.042**	0.847 ± 0.033	0.853 ± 0.050	0.837 ± 0.078
	4	**0.798** ± **0.046**	0.711 ± 0.048	0.692 ± 0.052	0.764 ± 0.040	0.757 ± 0.085	0.793 ± 0.054	**0.628** ± **0.074**	0.739 ± 0.060	0.751 ± 0.037	0.787 ± 0.131	0.752 ± 0.081
F1	1	0.674 ± 0.060	0.701 ± 0.037	0.690 ± 0.039	0.695 ± 0.054	0.661 ± 0.072	0.697 ± 0.108	**0.707** ± **0.049**	0.691 ± 0.057	0.698 ± 0.034	**0.638** ± **0.101**	0.707 ± 0.084
	2	0.844 ± 0.024	0.771 ± 0.030	0.841 ± 0.023	0.718 ± 0.048	**0.873** ± **0.026**	**0.712** ± **0.079**	0.797 ± 0.040	0.718 ± 0.065	0.811 ± 0.023	0.777 ± 0.074	0.814 ± 0.054
	3	0.765 ± 0.051	0.715 ± 0.048	0.691 ± 0.051	0.795 ± 0.045	0.733 ± 0.072	**0.806** ± **0.086**	**0.646** ± **0.076**	0.783 ± 0.073	0.723 ± 0.048	0.782 ± 0.073	0.720 ± 0.120
	4	0.669 ± 0.073	0.514 ± 0.081	0.508 ± 0.104	0.624 ± 0.063	0.610 ± 0.146	**0.687** ± **0.093**	**0.363** ± **0.169**	0.561 ± 0.089	0.599 ± 0.065	0.534 ± 0.217	0.577 ± 0.130

N.B. The values are mean ± SD of performance metrics from the RMFCV300 validation sets. The numbers in italics below each stratum name are mean ± SD of the sample sizes. For each TOAST for each performance metric, the largest and smallest mean values across the strata are highlighted in bold.

### Feature importance analysis

**Fig. 5:**
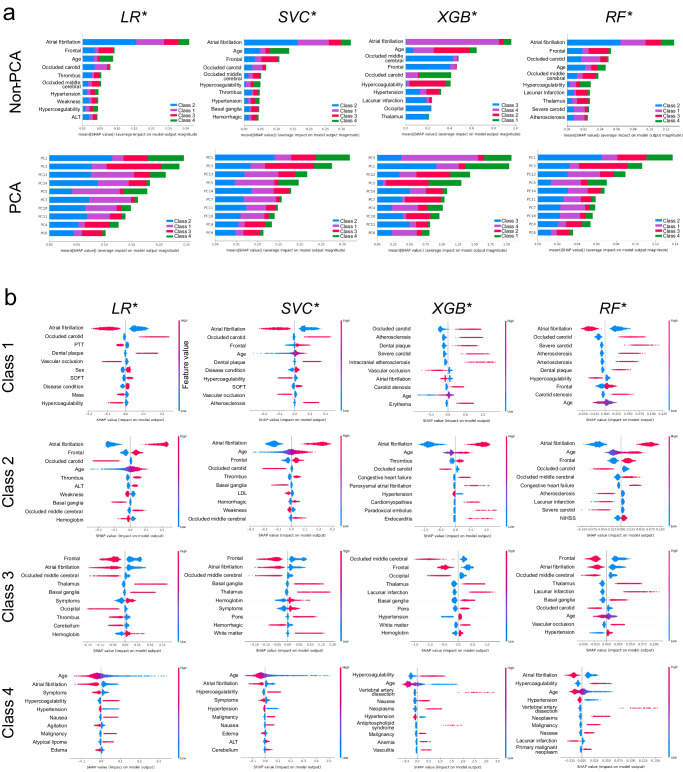
Feature importance by SHAP and statistical tests. **a** Top 10 features in terms of means of absolute SHAP values, mean( | SHAP | ), across all classes for each optimized model for non-PCA-based and PCA-based feature groups. **b** Top 10 features (non-PCA) in terms of SHAP values for each class for each optimized model.

We examined feature importance or the contribution of features to predict TOAST classification by SHAP analysis for each of the four optimized base models. The top ten features in terms of mean absolute SHAP values for each model are shown in Fig. 5a. The top feature for all four models is AF. The second feature is either the frontal location of the infarct noted on radiography or the patient’s age. For PCA, the top two features are PC1 and PC3 (the second and fourth principal components, respectively; 0-indexed). The largest impact of both AF and PC1 is on TOAST 2. We also examined the top ten features for each class for each model, as shown in Fig. 5b. The features that contribute the most to the prediction of TOAST 1 by all models were AF, carotid occlusion, and atherosclerosis; for TOAST 2 were AF, patient age, and frontal location of infarct; for TOAST 3 were frontal location of infarct, occluded middle cerebral artery, AF, and thalamus location of infarct; and for TOAST 4, patient age, AF, and hypercoagulability or thrombophilia. For the PCA-based optimized models, we examined the top five PCs and the top ten most contributing features for each PC for each class (Supplementary Fig. 4; Supplementary Table 15). Similar important features were observed, including age, sex, and NIHSS. This method identified multiple unique features contributing to stroke etiology classes. For example, the following six features in PC11 were unique to TOAST 2 by three models (SVC*, XGB*, and RF*): blood pressure (HEX), mass of body region (C0577573), Macrophage Activation Syndrome (C1096155), cyclic neutropenia (C0221023), sinus (HRT), and hemorrhagic (RAD). The following four features in PC10 are unique to TOAST 3 by three models (LR*, SVC*, and XGB*): left ventricular hypertrophy (HRT; C0149721), pericardial effusion (C0031039), and agitation (C0085631). The top features by the model-agnostic Kolmogorov–Smirnov test and Student’s *t*-test are largely in agreement, the correlations between |*t*| or *D* statistics (or their *p*-values) and means of absolute SHAP values averaged over the four models for the four classes ranging between 0.43 and 0.89 (Supplementary Fig. 5).

### Analysis of misclassification

**Fig. 6:**
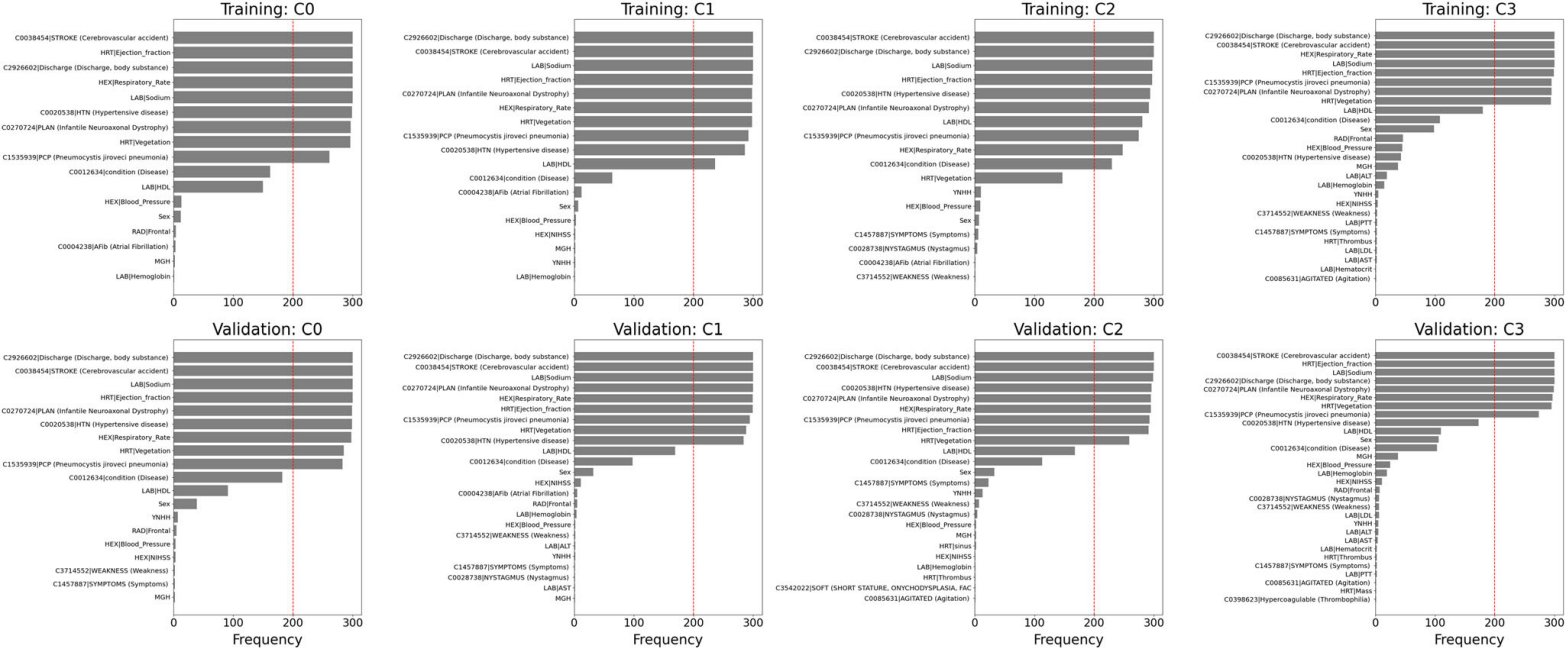
Top ten features of misclassification. Top ten features of misclassified samples for each class by the consensus model from RMFCV300.

We examined misclassified samples for each class and the top ten features of the highest frequency among those misclassified samples. We analyzed classification results by *StrokeClassifier* for both training and validation from the merged RMFCV300 results. The misclassification or error rates ( = 1 − accuracy; Supplementary Table 10) for training were 4.5 ± 0.6%, 5.3 ± 0.7%, 2.5 ± 0.4%, and 2.0 ± 0.4% for the 4 classes, respectively, and those for validation were 16.2 ± 1.4%, 16.8 ± 1.7%, 9.4 ± 1.2%, and 9.4 ± 1.2% for the 4 classes, respectively. The top 10 most frequent features among misclassified samples for each class in each training or validation set are found to be present in ≥54.8% of those samples (Supplementary Table 16). Frequencies of those top 10 features in the 300 training or validation sets for each misclassified class are shown in Table 5 and Fig. 6. There are 6 features that are among the top 10 in all of the 300 training or validation sets: cerebrovascular accident, ejection fraction, body substance discharge, respiratory rate, sodium, and infantile neuroaxonal dystrophy.

**Table 5:** Top ten features of the highest frequency for misclassification by *StrokeClassifier*

Misclassified etiology	Training		Validation	
	Top ten most frequent features	Frequency	Top ten most frequent features	Frequency
Large artery atherosclerosis (1)	C0038454 | STROKE (Cerebrovascular accident)	300	C2926602|Discharge (Discharge, body substance)	300
	HRT|Ejection_fraction	300	C0038454 | STROKE (Cerebrovascular accident)	300
	C2926602|Discharge (Discharge, body substance)	300	LAB|Sodium	300
	HEX|Respiratory_Rate	300	HRT|Ejection_fraction	300
	LAB|Sodium	300	C0270724 | PLAN (Infantile Neuroaxonal Dystrophy)	299
	C0020538 | HTN (Hypertensive disease)	299	C0020538 | HTN (Hypertensive disease)	299
	C0270724 | PLAN (Infantile Neuroaxonal Dystrophy)	297	HEX|Respiratory_Rate	298
	HRT|Vegetation	296	HRT|Vegetation	285
	C1535939 | PCP (Pneumocystis jiroveci pneumonia)	261	C1535939 | PCP (Pneumocystis jiroveci pneumonia)	283
	C0012634|condition (Disease)	162	C0012634|condition (Disease)	182
	LAB | HDL	150	LAB | HDL	91
	HEX|Blood_Pressure	13	Sex	39
	Sex	12	YNHH	7
	RAD|Frontal	4	RAD|Frontal	5
	C0004238|AFib (Atrial Fibrillation)	3	HEX|Blood_Pressure	3
	MGH	2	HEX | NIHSS	3
	LAB|Hemoglobin	1	C3714552 | WEAKNESS (Weakness)	2
			C1457887 | SYMPTOMS (Symptoms)	2
			MGH	2
Cardioembolism (2)	C2926602|Discharge (Discharge, body substance)	300	C2926602|Discharge (Discharge, body substance)	300
	C0038454 | STROKE (Cerebrovascular accident)	300	C0038454 | STROKE (Cerebrovascular accident)	300
	LAB|Sodium	300	LAB|Sodium	300
	HRT|Ejection_fraction	299	C0270724 | PLAN (Infantile Neuroaxonal Dystrophy)	300
	C0270724 | PLAN (Infantile Neuroaxonal Dystrophy)	298	HEX|Respiratory_Rate	299
	HEX|Respiratory_Rate	298	HRT|Ejection_fraction	299
	HRT|Vegetation	298	C1535939 | PCP (Pneumocystis jiroveci pneumonia)	294
	C1535939 | PCP (Pneumocystis jiroveci pneumonia)	292	HRT|Vegetation	288
	C0020538 | HTN (Hypertensive disease)	286	C0020538 | HTN (Hypertensive disease)	284
	LAB | HDL	236	LAB | HDL	169
	C0012634|condition (Disease)	64	C0012634|condition (Disease)	98
	C0004238|AFib (Atrial Fibrillation)	12	Sex	32
	Sex	7	HEX | NIHSS	11
	HEX|Blood_Pressure	3	C0004238|AFib (Atrial Fibrillation)	5
	HEX | NIHSS	2	RAD|Frontal	5
	MGH	2	LAB|Hemoglobin	4
	YNHH	2	HEX|Blood_Pressure	2
	LAB|Hemoglobin	1	C3714552 | WEAKNESS (Weakness)	2
			LAB | ALT	2
			YNHH	2
			C1457887 | SYMPTOMS (Symptoms)	1
			C0028738 | NYSTAGMUS (Nystagmus)	1
			LAB | AST	1
			MGH	1
Small vessel disease (3)	C0038454 | STROKE (Cerebrovascular accident)	300	C2926602|Discharge (Discharge, body substance)	300
	C2926602|Discharge (Discharge, body substance)	300	C0038454 | STROKE (Cerebrovascular accident)	300
	LAB|Sodium	298	LAB|Sodium	299
	HRT|Ejection_fraction	297	C0020538 | HTN (Hypertensive disease)	296
	C0020538 | HTN (Hypertensive disease)	294	C0270724 | PLAN (Infantile Neuroaxonal Dystrophy)	295
	C0270724 | PLAN (Infantile Neuroaxonal Dystrophy)	292	HEX|Respiratory_Rate	295
	LAB | HDL	281	C1535939 | PCP (Pneumocystis jiroveci pneumonia)	293
	C1535939 | PCP (Pneumocystis jiroveci pneumonia)	275	HRT|Ejection_fraction	291
	HEX|Respiratory_Rate	248	HRT|Vegetation	259
	C0012634|condition (Disease)	230	LAB | HDL	168
	HRT|Vegetation	147	C0012634|condition (Disease)	113
	YNHH	10	Sex	33
	HEX|Blood_Pressure	9	C1457887 | SYMPTOMS (Symptoms)	23
	Sex	7	YNHH	13
	C1457887 | SYMPTOMS (Symptoms)	6	C3714552 | WEAKNESS (Weakness)	7
	C0028738 | NYSTAGMUS (Nystagmus)	4	C0028738 | NYSTAGMUS (Nystagmus)	4
	C0004238|AFib (Atrial Fibrillation)	1	HEX|Blood_Pressure	2
	C3714552 | WEAKNESS (Weakness)	1	MGH	2
			HRT|sinus	2
			HEX | NIHSS	1
			LAB|Hemoglobin	1
			HRT|Thrombus	1
			C3542022 | SOFT	1
			C0085631 | AGITATED (Agitation)	1
			C0085631 | AGITATED (Agitation)	1
Other determined (4)	C2926602|Discharge (Discharge, body substance)	300	C0038454 | STROKE (Cerebrovascular accident)	300
	C0038454 | STROKE (Cerebrovascular accident)	300	HRT|Ejection_fraction	300
	HEX|Respiratory_Rate	300	LAB|Sodium	300
	LAB|Sodium	300	C2926602|Discharge (Discharge, body substance)	300
	HRT|Ejection_fraction	299	C0270724 | PLAN (Infantile Neuroaxonal Dystrophy)	299
	C1535939 | PCP (Pneumocystis jiroveci pneumonia)	295	HEX|Respiratory_Rate	297
	C0270724 | PLAN (Infantile Neuroaxonal Dystrophy)	295	HRT|Vegetation	295
	HRT|Vegetation	294	C1535939 | PCP (Pneumocystis jiroveci pneumonia)	274
	LAB | HDL	180	C0020538 | HTN (Hypertensive disease)	173
	C0012634|condition (Disease)	108	LAB | HDL	110
	Sex	98	Sex	106
	RAD|Frontal	46	C0012634|condition (Disease)	103
	HEX|Blood_Pressure	45	MGH	38
	C0020538 | HTN (Hypertensive disease)	43	HEX|Blood_Pressure	25
	MGH	38	LAB|Hemoglobin	19
	LAB | ALT	19	HEX | NIHSS	11
	LAB|Hemoglobin	15	RAD|Frontal	7
	YNHH	5	C0028738 | NYSTAGMUS (Nystagmus)	6
	HEX | NIHSS	4	C3714552 | WEAKNESS (Weakness)	6
	C3714552 | WEAKNESS (Weakness)	3	LAB | LDL	6
	LAB | PTT	3	YNHH	5
	C1457887 | SYMPTOMS (Symptoms)	2	LAB | ALT	5
	HRT|Thrombus	2	LAB | AST	4
	LAB | LDL	2	LAB|Hematocrit	2
	LAB | AST	2	HRT|Thrombus	2
	LAB|Hematocrit	1	C1457887 | SYMPTOMS (Symptoms)	2
	C0085631 | AGITATED (Agitation)	1	LAB | PTT	2
			C0085631 | AGITATED (Agitation)	1
			HRT|Mass	1
			C0398623|Hypercoagulable (Thrombophilia)	1

### Model generalizability by 5-way cross-hospital and longitudinal validation

To test the model generalizability, we applied the 9 base models (with **Χ(Λ**_**1**_**)**) to the curated MIMIC discharge summaries (Table 6). We used 3 versions of the MIMIC data as external validation: (1) MIMIC^0^ = 375 non-cryptogenic samples with 1406 features in common with YNHH and MGH, (2) MIMIC^1^ = 405 non-cryptogenic samples imputed by Random Forests using MICE, and (3) MIMIC^2^ = 405 non-cryptogenic samples imputed by random sampling using MICE. For MIMIC^1^, AUCROC ranged from 0.834 to 0.860 (0.847 ± 0.009), accuracy from 0.667 to 0.711 (0.691 ± 0.014), and F1 from 0.587 to 0.717 (0.690 ± 0.039) by the 9 base classifiers, while *StrokeClassifier* showed AUCROC of 0.809, AUPRC 0.719, accuracy of 0.699, F1 of 0.708, and kappa 0.467 (Table 6A). Performances in MIMIC^0^ and MIMIC^2^ or those by the PCA-based models were similar (Supplementary Table 17). Overall, the performance of *StrokeClassifier* in the external dataset was reduced by less than 5% in comparison with the internal 5-fold CV (Table 2B). We also examined class-wide performances of *StrokeClassifier* in MIMIC^1^. Prediction of TOAST 1 was associated with the lowest PPV of 37.0%, the lowest kappa of 0.377, and the highest false positive rate (FPR) of 11.4%; Prediction of TOAST 2 was associated with the lowest accuracy of 78.0%, the lowest F1 of 78.2%, the highest false negative rate (FNR) of 12.3%, the highest PPV of 84.1%, and the highest kappa of 0.535; Prediction of TOAST 3 was associated with the highest accuracy of 94.1%, the highest F1 of 94.6%, the lowest FPR of 4.0%, and the lowest FNR of 2.0%; performance measures for predicting TOAST 4 were moderate (Table 6B). Similar performances are observed for MIMIC^0^ and MIMIC^2^ (Supplementary Table 18).

**Table 6:** Model generalizability

(A) Global performances (weighted averages over all classes) on MIMIC by individual models							
Model	AUCROC	AUPRC	ACC	BA	PRC	F1	KAPP
LR*	0.834	0.750	0.679	0.614	0.735	0.694	0.444
SVC*	0.844	0.767	0.699	0.605	0.726	0.703	0.454
XGB*	0.860	0.783	0.711	0.614	0.752	0.717	0.483
RF*	0.843	0.779	0.667	0.461	0.725	0.587	0.251
SVC2	0.835	0.759	0.679	0.603	0.722	0.695	0.452
MAX	0.853	0.783	0.699	0.613	0.731	0.711	0.476
MIN	0.850	0.771	0.689	0.603	0.738	0.692	0.444
MEAN	0.854	0.781	0.701	0.613	0.734	0.710	0.471
MEDIAN	0.849	0.778	0.691	0.593	0.721	0.699	0.448
*Mean*	0.847	0.772	0.691	0.591	0.732	0.690	0.436
*SD*	0.009	0.012	0.014	0.049	0.010	0.039	0.071
*StrokeClassifier*	*0.809*	*0.719*	*0.699*	*0.608*	*0.735*	*0.708*	*0.467*

(B) Cross-hospital and longitudinal class-wide performances by *StrokeClassifier*								
Training data	Testing data	Class	Accuracy	PPV	F1	Kappa	FPR	FNR
YNHH + MGH (*N* = 1932)	MIMIC-RF (*N* = 405)	1	0.844	0.370	0.860	0.377	0.114	0.042
		2	0.780	0.841	0.782	0.535	0.096	0.123
		3	0.941	0.385	0.946	0.424	0.040	0.020
		4	0.842	0.689	0.831	0.475	0.047	0.111
MGH (*N* = 1002)	MIMIC-RF (*N* = 405)	1	0.849	0.369	0.862	0.357	0.101	0.049
		2	0.768	0.814	0.768	0.500	0.119	0.114
		3	0.956	0.500	0.956	0.477	0.022	0.022
		4	0.844	0.688	0.835	0.490	0.049	0.106
	YNHH (*N* = 930)	1	0.804	0.615	0.805	0.493	0.102	0.094
		2	0.803	0.760	0.804	0.600	0.108	0.089
		3	0.875	0.710	0.872	0.589	0.051	0.074
		4	0.891	0.533	0.891	0.465	0.053	0.056
YNHH (*N* = 930)	MIMIC-RF (*N* = 405)	1	0.820	0.296	0.837	0.267	0.123	0.057
		2	0.765	0.845	0.768	0.511	0.089	0.146
		3	0.923	0.314	0.935	0.379	0.059	0.017
		4	0.820	0.606	0.810	0.414	0.064	0.116
	MGH (*N* = 1002)	1	0.813	0.718	0.808	0.527	0.069	0.118
		2	0.821	0.767	0.822	0.637	0.105	0.074
		3	0.878	0.588	0.882	0.564	0.075	0.047
		4	0.888	0.589	0.886	0.502	0.051	0.061
Year 2015–2019 (*N* = 1688)	Year 2020 (*N* = 244)	1	0.852	0.733	0.851	0.611	0.066	0.082
		2	0.832	0.78	0.832	0.651	0.09	0.078
		3	0.893	0.727	0.895	0.687	0.061	0.045
		4	0.906	0.655	0.904	0.569	0.041	0.053

N.B. misclassification or error rate = 1 − accuracy; PPV = 1 − FDR (false discovery rate).

For an additional test of generalizability with **Χ(Λ**_**1**_**)**, we trained and optimized the four base models the same way as above using the MGH data of 1002 non-cryptogenic samples and applied to the YNHH and MIMIC data for external validation (Table 6B and Supplementary Table 18). The 4 best models, LR*_MGH_, SVC*_MGH_, XGB*_MGH_, and RF*_MGH_, yielded mean cross-validated AUCROC of 91.0%, 90.9%, 92.3%, and 91.1%, respectively, and accuracy of 74.4%, 73.6%, 76.8%, and 68.1%, respectively. The external validation of the YNHH and MIMIC^1^ data by *StrokeClassifier* resulted in an accuracy of 68.9% and 70.9%, respectively. Similarly, we next tested the models using the YNHH data of 930 non-cryptogenic samples for training and the MGH and MIMIC data for external validation (Table 6B and Supplementary Table 18). The 4 best models, LR*_YNHH_, SVC*_YNHH_, XGB*_YNHH_, and RF*_YNHH_, yielded mean cross-validated AUCROC of 86.8%, 86.5%, 87.6%, and 87.3%, respectively, and accuracy of 69.4%, 68.6%, 69.4%, and 60.6%, respectively. The external validation of the MGH and MIMIC^1^ data by *StrokeClassifier* resulted in an accuracy of 70.3% and 66.4%, respectively. Performances in MIMIC^0^ and MIMIC^2^ were similar (Supplementary Table 18).

To address the longitudinal useability of StrokeClassifier, we re-trained and optimized the model with a new training set of discharge summaries from 2015 to 2019 in the combined cohort of YNHH and MGH and then longitudinally validated the optimal model using a test set from 2020. The performances are AUCROC of 86.8%, AUPRC of 71.4%, accuracy of 74.2%, F1 of 74.0%, and Cohen’s kappa of 0.64 for multi-class classification. For binary classification of each of the 4 TOAST classes, accuracy and F1 range from 83.2% to 90.6% (Table 6B).

### Predicting etiologies of cryptogenic stroke using StrokeClassifier

**Fig. 7:**
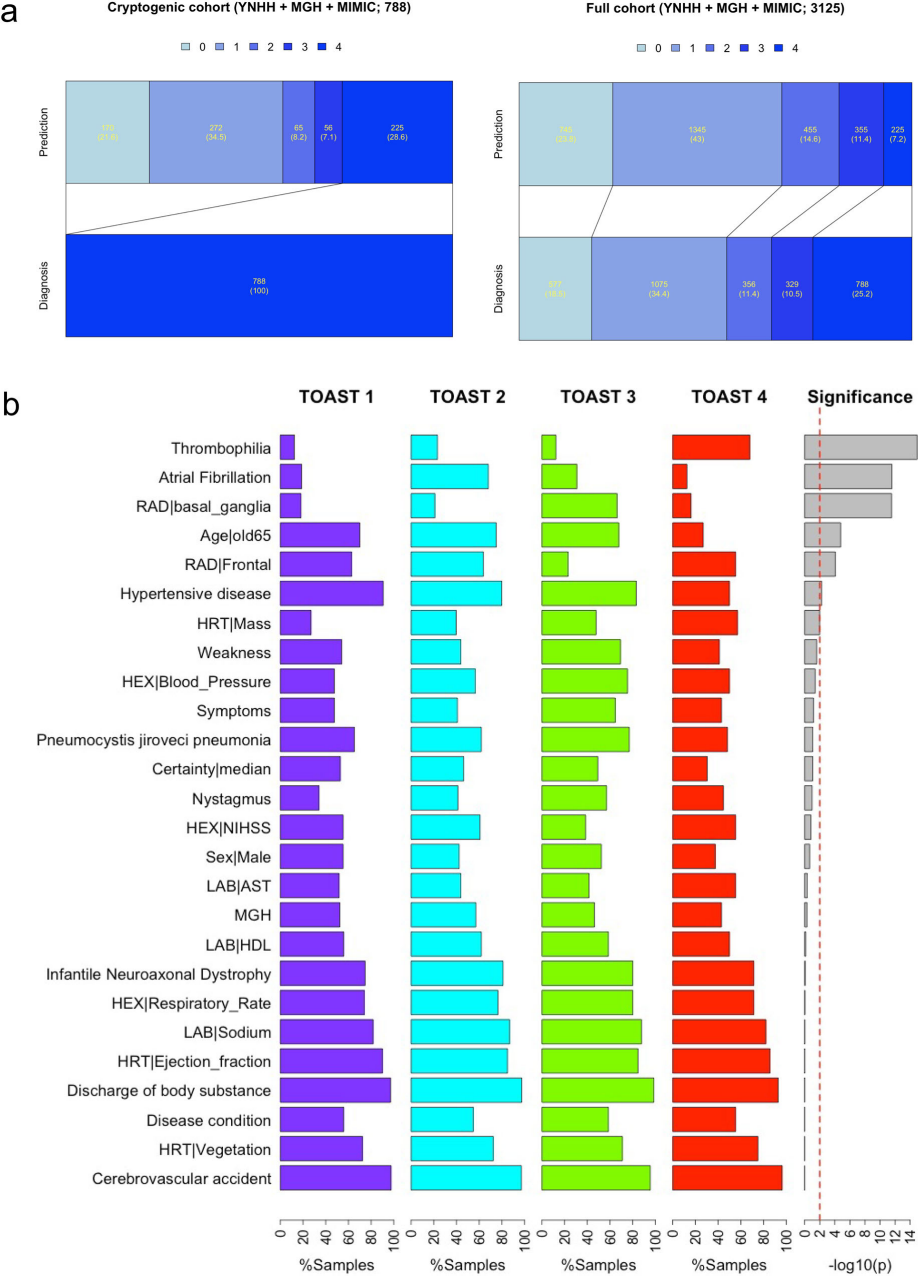
Prediction of cryptogenic samples and highly frequent features for each predicted class. **a** The bar graphs show a prediction distribution of all cryptogenic patients by StrokeClassifier (left) and a resultant prediction distribution of all of non-cryptogenic and cryptogenic patients (right). **b** The bar plots show class-wide frequency distributions of highly frequent features. There are 26 features which are present in >50% of those cryptogenic samples of any predicted TOAST class. The significance was tested by chi-squared tests.

We next aimed to classify a potential etiology of strokes in a cohort of adjudicated cryptogenic strokes using a variety of certainty heuristics as proof-of-concept. In the pooled cohort of YNHH, MGH, and MIMIC^1^ datasets, there were a total of 788 stroke patients (285, 409, and 94, respectively), which were deemed to be cryptogenic strokes by vascular neurologists (Table 7). The heuristic that we employed in this study was built on a threshold of the first quartile (25% or moderate confidence) of the number of consensus supports among the 9 base classifiers for each TOAST classification based on the MIMIC^1^ external validation results: 7 supports for TOAST 1, 9 for TOAST 2, 7.2 for TOAST 3, and 7 for TOAST 4 (Supplementary Table 19). If the number of supports for a particular sample was greater than or equal to the prespecified TOAST class threshold, the ischemic stroke was classified as the corresponding TOAST class. If the number of supports was less than any of the pre-specified TOAST class thresholds, the etiology was classified as persistently cryptogenic. Table 7 shows distributions of predicted TOAST classifications of cryptogenic patients for each cohort and the pooled cohort. Figure 7a also depicts the distributions of TOAST classification of the full cohort as adjudicated by vascular neurologists versus *StrokeClassifier*. Predictions for 46.3%, 54.5%, and 37.2% of the cryptogenic samples of YNHH, MGH, and MIMIC^1^ were agreed by all the 9 base classifiers, respectively. The prediction agreement by at least 8 base classifiers was observed for 69.8%, 72.6%, and 61.7% of the cryptogenic samples of YNHH, MGH, and MIMIC^1^, respectively. The most frequently predicted etiology was TOAST 2 for YNHH and MGH (32.6% and 37.9%, respectively) and TOAST 1 for MIMIC^1^ (27.7%), whereas the least frequently predicted etiology was TOAST 4 for YNHH and MGH (6.7% and 5.9%, respectively) and TOAST 3 for MIMIC^1^ (5.3%) (Table 7). The percentages of persistently cryptogenic samples for YNHH, MGH, and MIMIC^1^ were 30.9%, 27.1%, and 27.7%, respectively (Table 7). In other words, 28.6% of all cryptogenic samples (225 out of 788) were not predicted with high confidence by *StrokeClassifier* and remain cryptogenic. This reduced the percentage of cryptogenic patients from 25.2% to 7.2% in the full cohort of 3125 stroke patients in YNHH, MGH, and MIMIC (Fig. 7a). In contrast, when we used a certainty heuristic of the third quartile number of consensus supports (high confidence), 9.9% of cryptogenic patients (309 cryptogenic patients of the full cohort; Supplementary Table 19) remained persistently cryptogenic.

**Table 7:** Application of *StrokeClassifier* to cryptogenic stroke patients

TOAST predicted	YNHH	MGH	MIMIC (RF-imputed)	Merged
1	55 (19.3%)	89 (21.8%)	26 (27.7%)	170 (21.6%)
2	93 (32.6%)	155 (37.9%)	24 (25.5%)	272 (34.5%)
3	30 (10.5%)	30 (7.3%)	5 (5.3%)	65 (8.2%)
4	19 (6.7%)	24 (5.9%)	13 (13.8%)	56 (7.1%)
Persistently cryptogenic	88 (30.9%)	111 (27.1%)	26 (27.7%)	225 (28.6%)
Total	285 (100%)	409 (100%)	94 (100%)	788 (100%)

Finally, we generated a repertoire of EHR signatures of predicted TOAST classes for cryptogenic strokes (excluding the 225 persistently cryptogenic strokes) using feature frequencies from *StrokeClassifier*. We focused on those features that were present in >50% of the cryptogenic stroke samples in each predicted class. We identified 26 such features (Fig. 7b). Six of these 26 features were class-specific with *p*-value < 0.01 by chi-squared tests: hypercoagulability/thrombophilia (high-frequency for TOAST 4; *p* = 1.19e−15), AF (high-frequency for TOAST 2; *p* = 2.69e−12), basal ganglia (high-frequency for TOAST 3; *p* = 2.93e−12), age >65 (low-frequency for TOAST 4; *p* = 1.68e−05), frontal (low-frequency for TOAST 3; *p* = 8.60e−05), and hypertensive disease (low-frequency for TOAST 4; *p* = 5.66e−03).

## Discussion

We developed and validated an accurate automated tool, *StrokeClassifier*, to predict AIS etiology using EHR text-based data collected during stroke hospitalization. *StrokeClassifier* is a meta-classifier of a majority voting ensemble built from nine base classifiers trained using adjudicated outcomes curated from institutions with vascular neurology expertise. Standardized CUI features extracted from unstructured or semi-structured text corpora by an NLP method were particularly powerful predictors. We found that the predictive capacity of *StrokeClassifier* was generalizable in five-way external validation cohorts as well as a longitudinal analysis. While limited in several ways, this work is a promising multi-cohort and multi-class study of stroke subtype classification. The external and longitudinal validation accuracies were about 70% and 74%, respectively, for multi-class classification, while they were 77–96% for binary classification. These accuracies are higher than the minimum accuracy of 70% desired by a convenience sample of 13 international clinicians who care for stroke patients to adopt an AI stroke etiology diagnostic tool into clinical practice (8 vascular neurologists, 3 non-vascular neurologists, and 2 internists who we interviewed during the National Science Foundation Innovation Corps Regional Program, Summer 2023). By applying *StrokeClassifier* to a cohort of cryptogenic stroke patients to predict non-cryptogenic stroke etiologies with a certainty heuristic, the proportion of ischemic stroke patients in the full cohort with a persistently cryptogenic diagnosis was 7.2%, which was 71% lower than the rate adjudicated by vascular neurologists. With further training in representative cohorts, *StrokeClassifier* may aid stroke etiology diagnosis during the stroke hospitalization and timely administration of secondary stroke prevention therapies. It may also inform future clinical and population research investigations.

There are three published manuscripts and one abstract describing machine learning classifiers for ischemic stroke TOAST classification subtyping with various limitations that we aimed to overcome^27,28,32^. Inclusion criteria for specific stroke etiologies varied in these studies with downstream implications. The studies by Garg et al. and Turner et al. trained models to classify all 5 TOAST subtypes^27,32^, while the study by Wang et al. excluded cryptogenic strokes altogether^29^. Sung et al. explored multiple machine learning classifiers and MetaMap for multi-class classification of the 4 Oxfordshire Community Stroke Project subtypes of ischemic stroke using admission clinical notes from a single cohort, but none of their classifiers exceeded an accuracy of 60%^34^. Kamel et al. trained a binary classifier using non-cryptogenic stroke samples and then applied the classifier to cryptogenic stroke samples^28^. We utilized a stepwise approach, with the goal of ultimately classifying subtypes. We did not consider cryptogenic samples during training because they were comprised of a mixture of potential etiologies^36^. Instead, we investigated distributions of the 4 predicted non-cryptogenic etiologies for cryptogenic samples. We then developed various certainty heuristics to predict the probability of stroke etiologies, both non-cryptogenic and persistently cryptogenic. This scalable property of *StrokeClassifier* is promising since the patients it is tasked to classify will not be pre-specified as cryptogenic or non-cryptogenic. All published stroke etiology classifiers were trained and tested at a single center, which may not generalize to other centers in the U.S. or globally^27–29,32^. *StrokeClassifier* was tested in separate hospital cohorts with various EHR systems, and robustness was demonstrated. Each classifier, with the exception of the one developed by Garg et al., relied on hard-coded fields and did not have the capacity to utilize unstructured text data. Although the classifier generated by Garg et al. applied natural language processing to text-based data, it lacked an established ontological framework that could map phraseologies to consistent clinical concepts. We leveraged the UMLS conceptual framework developed by the National Library of Medicine to ensure the operability of *StrokeClassifier* irrespective of clinician and computer environment. For computational efficiency, we utilized PCA to capture multi-dimensional contributions of a wide array of features. We uniquely trained *StrokeClassifier* on adjudicated stroke etiologies upon review by at least two board-certified vascular neurologists. Since there was variability among individual optimized models in predicting each etiology, the 4 optimized models, along with SVC2, were aggregated into ensemble models, which are also architecturally simple and efficient. Although ensemble modeling was utilized by Kamel et al.^28^ it did not include the diversity of models that *StrokeClassifier’s* meta-model represents with summary-statistic-based ensemble models. We took several measures to minimize bias. To address overfitting, we investigated sub-optimal models within 1 standard deviation of the optimized models in terms of AUCROC, showing performance reduction by up to 4% across different metrics and CV folds. Additionally, in an effort to offset bias introduced by relying on a single choice of CV folds and a particular random seed, our RMFCV300 strategy analysis offers a more robust framework to assess model performance and generalization errors. Finally, we performed SHAP analyses to assess the degrees to which features contributed to stroke etiology prediction. The features contributing to the prediction of each stroke etiology were biologically plausible, lending validity to *StrokeClassifier*.

There are multiple potential applications of a trained, automated, accurate, and computationally efficient stroke etiology classifier. It can be implemented in health systems to perform the complex task of synthesizing the copious, semi-structured data collected during an AIS hospitalization and rapidly classifying the underlying stroke etiology in an automated manner for millions of patients. Most proximally, automated stroke etiology prediction can cue a treating clinician to consider instituting a targeted treatment by reducing diagnostic uncertainty and diagnostic errors due to human cognitive biases, oversight, and therapeutic inertia^37^. In healthcare settings where vascular neurology expertize is sparse or unavailable, *StrokeClassifier* may be especially valuable^23^. A classifier such as *StrokeClassifier* can be harnessed by informaticians to create nudges or progress notes indicating predicted etiologies and guideline-recommended therapies for individual patients. Stroke etiology data fields collected by manual extraction are currently incomplete in registries in the U.S. at all levels and, when populated, are often inaccurate as seen in our study. Stroke etiology predictions can be linked to institutional, regional, and country-wide registries to facilitate quality improvement, clinical trials, public health, and health services research efforts. Finally, it may identify patients with established stroke etiologies and risk factors which may render them eligible for clinical trials studying alternative secondary stroke prevention therapies.

While the *StrokeClassifier* was trained with the task of classifying etiology at the time of discharge, the predictive factors identified may be collected at an earlier timepoint during the hospitalization. The classifier was trained using data collected during the course of the AIS hospitalization and populated into the discharge summary, which is typically finalized at the completion of the hospital encounter. We observed that the sources of information that contributed most to the model’s diagnostic performance individually and in our leave-one-out analysis in descending order as presented in Table 2 were (1) concept unique identifiers or CUIs (AUCROC range: 0.87–0.89), (2) radiologic features of neuroanatomic location of the ischemic stroke, vessel patency, and hemorrhagic transformation (AUCROC range: 0.76–0.77), and (3) cardiac features from electrocardiographic and echocardiographic reports (AUCROC range: 0.61–0.63). While CUIs represent a baseline medical history, conventional neuroimaging such as computed tomography with angiography and electrocardiograms are collected at the time of presentation during an acute stroke code, other data such as diagnoses accrued during the stroke hospitalization encounter, advanced neuroimaging such as magnetic resonance imaging, and cardiac imaging including echocardiography are typically obtained during later timepoints, if at all, depending on the resources and level of expertize housed within a healthcare setting. Future studies are necessary such as transfer learning of *StrokeClassifier* to a new task of classifying stroke etiology using solely data collected on the day of stroke presentation. One potential implication of the heavy reliance of model performance on CUIs, radiologic features, and cardiac features is that a clinical decision support tool may be designed to prompt a recommendation to order diagnostic evaluations associated with missing feature categories to improve stroke etiology prediction.

The capacity to predict an underlying etiology of cryptogenic strokes using *StrokeClassifier* is promising. The predicted etiology among cryptogenic patients in the YNHH and MGH cohorts was predominantly cardioembolism, varying from 33% to 38%, followed by large artery atherosclerosis in 19% to 22%. Secondary analysis of the NAVIGATE ESUS study demonstrated that among ESUS patients, there were multiple potential etiologies, including atrial cardiopathy (37%), left ventricular disease (36%), and arterial atherosclerosis (29%), with no potential etiology found in only 23% of patients and more than 1 potential etiology in 41% of patients^36^. Given that many cryptogenic stroke patients have multiple potential sources, applying an algorithm such as *StrokeClassifier* can be especially fruitful because its supervised learning of features that may non-linearly associate with etiologies may be transferable. *StrokeClassifier* is a majority-voting consensus prediction tool from multiple base classifiers. We harness this property to address the uncertainty that arises when a patient has multiple competing potential sources of stroke. This is represented by the *StrokeClassifier* assigning confidence levels in terms of the degree of agreement among the base classifiers, a construct we denote as a certainty heuristic. When the number of individual classifiers voting for two potential etiologies is equal for a patient, the patient’s etiology is classified as cryptogenic due to uncertainty. This computational decision-making process is analogous to the diagnostic process used by human clinicians, who deem an etiology to be cryptogenic when the probabilities of multiple etiologies are equally likely^3^. To provide interpretability in instances when an etiology is deemed cryptogenic due to multiple potential sources, the output of *StrokeClassifier* can include voting results of the individual classifiers so that the user is informed about the percentage of classifiers that voted for a particular etiology (e.g., Supplementary Table 20 for the MIMIC data). Further work is needed with probabilistic techniques to parse out stroke etiopathogenesis in patients with multiple etiologies. It also remains to be determined whether implementing therapies targeting all likely etiologies at the time of an AIS hospitalization may be superior to the standard of care.

We derived EHR signatures corresponding to the predicted etiology of cryptogenic stroke patients. It begins to provide a conceptual and workflow framework for strokes traditionally deemed cryptogenic. For instance, cryptogenic patients with predicted etiology of large artery atherosclerosis by *StrokeClassifier* tend to be older and have frontal infarct, hypertension, and no AF. Thus, predicted stroke etiology classification of patients with these features during stroke hospitalization may prompt deeper, streamlined inquiry into this potential mechanism, such as more advanced vascular imaging to assess the characteristics of a sub-stenotic carotid plaque. It may also obviate the need for broad, unnecessary testing that leads to health care expenditure. Predictions may also make clinicians uncertain about which of multiple competing etiologies led to the stroke in a singular direction. This information and subsequent diagnostic investigation may then lead to the initiation of evidence-based targeted secondary stroke prevention therapy. Finally, in an era of biomarker-based clinical studies, the potential stroke etiology signatures yielded by classifiers such as *StrokeClassifier* may advance research by identifying an enriched population of cryptogenic ischemic stroke patients who may benefit from specific trial interventions for secondary stroke prevention.

Our study has limitations. The scope of this study was limited by its cross-sectional design; our future goal is to further train *StrokeClassifier* in longitudinal cohorts to enable it to predict the eventual etiologic diagnosis in patients initially deemed cryptogenic. While the gold standard method of discerning stroke etiology is based on pathologic confirmation, an invasive procedure such as a brain biopsy is exceedingly rare. Thus, our outcome measure, while adjudicated by vascular neurology specialists, is ultimately probabilistic. Although training occurred using data from two academic institutions which are Comprehensive Stroke Centers, there was notable variability in clinical documentation and degree of testing by site as well as in prediction performances (Tables 1 and 6B). Nevertheless, training *StrokeClassifier* in this heterogeneous environment ensured generalizability across clinician training and documentation styles, EHR systems, and formatting. Further training in other cohorts is needed to increase the capture of more features. The epidemiology of stroke etiology may differ by geographic region, race, or ethnicity, and prevalence may impact predictive accuracy^38^. This study spanned the time period before and during the COVID-19 pandemic. We demonstrated previously that the distribution of TOAST subtypes of ischemic stroke etiology was similar before and during the COVID-19 pandemic at YNHH^39^. Finally, despite the identification of optimal models via HPO, there remains room for further exploration of other hyperparameters.

In conclusion, we present *StrokeClassifier*, a validated diagnostic tool developed using an innovative modeling strategy that allows automated, real-time classification of stroke etiology in an accurate and computationally efficient manner with EHR text data inputs. Its immediate application may be as a clinical decision support tool to aid in the diagnosis of stroke etiology, prompting targeted secondary stroke prevention therapies in a timely manner. Furthermore, the *StrokeClassifier* may facilitate the abstraction of stroke etiology in population-based registries to aid epidemiologic, health policy, and clinical research efforts.

## Methods

### Study population and data sources

The derivation cohort consisted of hospitalizations at two academic Comprehensive Stroke Centers of Yale New Haven Hospital (YNHH) and Massachusetts General Hospital (MGH) from 2015 to 2020. Institutional Review Board approval was obtained from both YNHH and MGH. The external validation cohort was a subgroup of hospitalizations at the academic Comprehensive Stroke Center of Beth Israel Deaconess Medical Center from 2001 to 2012. Access to this cohort’s data was obtained through the MIMIC-III (Medical Information Mart for Intensive Care) warehouse, which contains records of 46,520 hospitalizations from 2001 to 2012 at Beth Israel Deaconess Medical Center. MIMIC-III is a publicly available, de-identified health record repository that was developed and approved by the Beth Israel Deaconess Medical Center and Massachusetts Institute of Technology IRBs^40^. Two of the authors (H.L. and R.S.) were approved to have access to this database for research after passing the requisite training course^40,41^.

Acute ischemic stroke hospitalizations at YNHH and MGH were identified by each institution’s Get-with-the-guidelines stroke database. Get-With-The-Guidelines (GWTG)-Stroke database is a quality improvement initiative in which participating hospitals enter clinical and radiographic data of all patients hospitalized with an ischemic stroke diagnosis^42^. Acute ischemic stroke patients are identified by administrative billing codes (International Classification of Diseases (ICD), 10th Revision). Data abstraction, entry, and adjudication are performed by trained study personnel. There are logic checks and form controls to minimize data entry errors. The database was queried for all ischemic stroke patients ≥18 years admitted from January 2015 to December 2020 at MGH and YNHH to assemble the ischemic stroke cohort. The EHR platform for both institutions is Epic (Epic Systems Corporation), the most prevalent EHR system in the United States. Stroke hospitalizations from the GWTG databases were linked with corresponding semi-structured discharge summary plain ASCII text files, resulting in a total of 1269 and 1493 records from YNHH and MGH, respectively.

The MIMIC-III dataset was queried for the ICD-9 codes of 433.X and 434.X that are associated with ischemic stroke, resulting in a total of 2563 hospitalization records from patients ages >18 years admitted to BIDMC from 2001 to 2012. A subset of these, a convenience sample of the first consecutive 500 records, were included in this study for external validation and their discharge summary plain ASCII text files were analyzed. BIDMC utilizes its own customized, hospital-wide EHR system. A description of the study populations from the three institutions represented in this analysis is provided in Table 1.

### Outcomes

The primary study outcome was stroke etiology as defined by the five mutually exclusive causative mechanisms of stroke per the TOAST classification system: 1—large artery atherosclerosis, 2—cardioembolism, 3—small vessel disease, 4—other determined etiology, and 5—undetermined etiology (cryptogenic)^3^. Stroke etiology was determined by the agreement of two board-certified vascular neurologists. The first vascular neurologist was the discharging treating clinician, when applicable, who documented a stroke etiology impression in the EHR. The second vascular neurologist was the study co-author (R.S.), who reviewed the entire stroke hospitalization record and viewed the neuroimaging. When either there was disagreement about the stroke etiology between the two vascular neurologists or the discharging treating clinician was not a vascular neurologist (4% and 2% of the YNHH and MGH cohort, respectively), a third vascular neurologist at each of the two institutions (A.D. and A.C.T. at YNHH and MGH, respectively) reviewed the entire stroke hospitalization record and provided stroke etiology diagnosis impressions. The final stroke etiology diagnosis was the etiology ascribed by the majority. If there was no majority, the stroke etiology diagnosed by the senior-most vascular neurologist was utilized. In the external validation cohort, the co-author, R.S., reviewed the text of each discharge summary and designated a TOAST classification based on the data recorded in the text corpus.

### Covariates

(a) *Demographic variables*. Using regular expressions, we extracted age and sex from the discharge summary text. The YNHH dataset did not contain sex information in a structured format in the discharge summary, unlike the MGH data. To identify sex information from the YNHH data, we used a customized R code to search for “her” or “his” in the EHR texts to assign female or male to each EHR, respectively. We compared the accuracy of this extraction with the age and sex fields hardcoded in the corresponding institutional GWTG-stroke registry. We intentionally did not include the proxy variable of race as a covariate for model training and testing because our datasets lack measures of the social environment which may be more relevant indicators of stroke etiology than ancestry alone^43^.

(b) *Clinical variables derived from MetaMap*. We applied natural language processing tools to the corpus of discharge summary texts to engineer clinical variables that may be associated with stroke etiology. Firstly, discharge summaries were processed by the natural language processing (NLP) or text mining tool, *MetaMap*, developed by the National Library of Medicine (NLM) to extract terms from text and link them to standard biomedical concepts in the Unified Medical Language System (UMLS) Metathesaurus^44,45^. Each discharge summary is a semi-structured text that can be processed by MetaMap to detect unique concepts or concept unique identifiers (CUIs) from the UMLS, which contains over 1 million biomedical concepts in an automated manner. We applied MetaMap to the discharge summary text of each hospitalization and extracted CUIs that belong to the following three types or categories: “Disease or Syndrome”, “Neoplastic Process”, and “Sign or Symptom” (Supplementary Table 1). The rationale for selecting MetaMap CUIs was that it was designed to retrieve medical concepts by lexical analysis and tokenization. MetaMap allows for abbreviations, acronyms, negations, and parts-of-speech tagging. It facilitates lookups in the SPECIALIST system that is supported by the UMLS Metathesaurus and Semantic Network, a repository of biomedical concepts and their interrelationships^46^ that is updated quarterly and incorporates SNOMED CT content which is routinely utilized in SNOMED CT-enabled EHR systems to enable meaning-based retrieval of information and maps to ICD-9 and ICD-10 coding systems^47^. MetaMap also performs word sense disambiguation by which concepts are favored if semantically consistent with the surrounding text. There is also flexibility in input and output data formats permissible by MetaMap. Finally, MetaMap has been rigorously tested in various biomedical research applications^48,49^. Compared with other clinical entity extraction tools, MetaMap was demonstrated to have the highest recall and F1 score when tasked with identifying clinical concepts such as obesity-related symptoms^50^. In one study, MetaMap extracted biomarker types from pathology reports with >95% accuracy^51^.

(c) *Other variables*. By employing customized regular expressions, we curated four other categories of features from discharge summaries (Supplementary Table 2). First, we extracted clinical information not captured by CUIs, including social history (tobacco, ethanol, and illicit drug use), National Institutes of Health Stroke Severity scale, and vital signs, which we designate as six HEX features. Second, we extracted 40 radiologic features (RAD) from studies performed during the stroke hospitalization, including information about the neuroanatomical location of the ischemic stroke, the presence of moderate or severe stenosis or occlusion of specific head and neck arteries, and the occurrence of intracranial hemorrhage encoded as a binary variable. The accuracy of our automated method of radiology data extraction in a random sample of 100 selected for each variable was 98% for neuroanatomic location and 99% for vessel abnormality^52^. Third, we also extracted 36 cardiac features (HRT) from electrocardiography and echocardiography reports in the discharge summary. Finally, we extracted 18 laboratory features (LAB). All lab values were generated during the stroke hospitalization encounter. In a random sample of 5 YNHH and 5 MGH patients, the accuracy of the HRT and LAB features that were extracted was 100%. In order to reduce measurement noise or error, we discretized the continuous values of the HEX and LAB features into clinically relevant categories. Ejection fraction was dichotomized as <40% which is defined as severely reduced versus ≥40%^53^, NIHSS was dichotomized as <6 defining a minor stroke versus ≥6^54^, sodium level < 136 mmol/l which is defined as hyponatremia^55^ versus >= 136 mmol/liter, BUN > = 24 mg/dL which is the upper limit of its normal range^56^ including in the elderly versus < 24 mg/dL and per the clinical laboratories of Yale and MGH, ALT and AST < 36 U/L versus ≥36 U/L per the clinical laboratory of Yale (https://www.ucsfhealth.org/medical-tests/alanine-transaminase-(alt)-blood-test#), white blood cell count < 11 × 1000/µl versus ≥11 × 1000/µl which defines leukocytosis^57^ and per the clinical laboratories of Yale and MGH, hematocrit < 35% (anemia), 35–45% (normal), ≥46% (erythrocytosis) per Yale and MGH clinical laboratories, hemoglobin in females < 11.7 (anemia), 11.7–15.5 (normal), and >15.5 (erythrocytosis) per Yale’s clinical laboratory, hemoglobin in males <13.2 g/dL (anemia), 13.2–17.1 g/dL (normal), and >17.1 g/dL (erythrocytosis) per Yale’s clinical laboratory, triglyceride ≥ 200 mg/dL which defines hypertriglyceridemia^58^ and per Yale and MGH clinical laboratory versus <200 mg/dL, HDL mg/dL < 40^59^ versus ≥40 mg/dL, LDL ≥ 100 mg/dL^60^ versus <100 mg/dL, TSH < 4.2 micro IU/mL versus ≥4.2 micro IU/mL^61^, PTT < 29.9 versus ≥30 s per Yale clinical laboratory, and hemoglobin A1c ≥ 6.5% which defines diabetes^62^ versus <6.5%. We denote the discretized feature groups by HEXd and LABd. We assess model performance based on each of the five feature groups, all the five groups, or those five combinations excluding each group. We assess the completeness of the investigation for stroke etiology during hospitalization based on values available for each of these groups.

### Imputation of missing data

We deployed a multiple imputation method, MICE (multivariate imputation by chained equations)^63,64^, from the *mice* package in R to impute missing values in categorical and numerical features of the YNHH and MGH data using the built-in method of predictive mean matching (*pmm*) with the default parameters. We also imputed the missing MIMIC features using the built-in method of Random Forests (*rf*; with the default parameters), which we found was better for dealing with larger fractions of missing values than *pmm* or other built-in imputation methods.

### Dimensionality reduction of features by principal component analysis

Since the number of features totaled 2027, we explored the relationship between dimensionality reduction of features and model training and performance. We chose principal component analysis (PCA) to reduce the feature dimensionality because of its clear interpretation of each principal component as a linear combination of all features. We applied PCA to all features and selected the top PCs for each of the following 10 thresholds of the total variance: 10%, 20%, 30%, 40%, 50%, 60%, 70%, 80%, 90%, 95%, and 99%. Validation and test datasets were transformed based on PCA of training datasets.

### Machine learning model development and evaluation

We analyzed non-cryptogenic ischemic stroke hospitalization records of discharge summaries from the merged YNHH and MGH datasets for model training and internal cross-validation. Figure 1 shows an overview of our workflow. Records from non-cryptogenic ischemic stroke hospitalizations in the MIMIC dataset were used as the test dataset (i.e., for external validation). We built models using the following 20 different feature groups individually: CUIs; RAD; HRT; HEX; HEXd; LAB; LABd; RAD + HRT + HEX + LAB, CUIs + HRT + HEX + LAB, CUIs + RAD + HEX + LAB, CUIs + RAD + HRT + LAB, CUIs + RAD + HRT + HEX, CUIs + RAD + HRT + HEXd, CUIs + RAD + HRT + HEX + LAB, and CUIs + RAD + HRT + HEXd + LABd. For the last two groups, we also applied filtering of samples based on maximum information (MaxInfo) ≥ 4 (the number of feature categories present) and the 11 PCA-based feature groups described above.

We built base models using four different supervised machine learning algorithms to classify the four-level non-cryptogenic stroke etiology outcome: logistic regression (LR), support vector classifier (SVC), Random Forests (RF), and XGBoost (XGB). Each model was optimized with a grid search of a pre-defined hyperparameter space for each of 24 training datasets, i.e., a total of 96 ( = 4*24) hyperparameter optimization (HPO) runs, and a stratified cross-validation (CV) strategy of 5 splits of 20% validation sets using *StratifiedShuffleSplit* from the *scikit-learn* library in Python. We controlled the randomness of the stratified CV by setting the parameter *random_state* = 1701 in this work. The best models with optimized parameters were selected based on the maximum AUCROC (the area under the curve of the receiver operating characteristic). Mathematical representations of a classifier, ${\psi }_{m}$, are as follows:

1 $$\begin{array}{l}{\varPsi }_{m}\left({{\mathcal{H}}}_{{\psi }_{m}},{{{{\rm X}}}}_{\alpha ,{\beta }_{l}}\right)={{{\omega }}}_{k}\\ \Psi =\left\{{\varPsi }_{m}\!:{\rm{classifiers}},\,m=1,2,\ldots, M\right\}{{;}}\,M={\rm{||}}\Psi {{||}}\\ {{\mathcal{H}}}_{{\psi }_{m}}=\left\{h\!:{\rm{hyperparameters}}\right\}\\ {{{{\rm{X}}}}}_{\alpha ,{\beta }_{l}}=\{\alpha \in \left\{{\rm{samples}}\right\},{\beta }_{l}\,{{\epsilon }}\,{\Lambda }_{l}=\left\{{\rm{features}}\right\}\,:\alpha =1,2,\ldots, N{;}\,{\beta }_{l}=1,2,\ldots, {L}_{l}\}\\ \Lambda =\left\{{\Lambda }_{l}=\left\{{\rm{features}}\right\}\!:l=1,2,\ldots, Q\right\}{;}\,Q={\rm{||}}\Lambda {{||}}\\ \Omega =\left\{{\omega }_{k}\!:{\rm{classes}\; or\; labels},k=1,2,\ldots, K\right\}{{;}}\,K={{||}}\Omega {{||}},\end{array}$$

where *M* = 4 classifiers (LR, SVC, RF, XGB), *N* = 2626 samples, *max(L*_*l*_*)* = *2027* features, *Q* = 20 feature groups, and *K* = 4 TOAST classes. The detailed configurations for HPO of the 4 classifiers are as follows:

(a) LR: We used *LogisticRegression* from the *sklearn* library in Python. The following parameter values were used for a grid search of 143 combinations with penalty = ‘elasticnet’ (elastic net, lasso, or ridge regularization), the saga solver, and 500 max iteration: *C* = (1e − 2, 1e − 1, 1e + 0, 1e + 1, 1e + 2, 1e + 3, 1e + 4, 1e + 5, 1e + 6, 1e + 7, 1e + 8, 1e + 9, 1e + 10) and l1_ratio = (0.0, 0.1, 0.2, 0.3, 0.4, 0.5, 0.6, 0.7, 0.8, 0.9, 1.0). The optimized parameters are *C* = 0.01 and l1_ratio = 0.0.

(b) SVC: We used SVC from the *sklearn* library in Python. The following parameter values were used for a grid search of 676 combinations with decision_function_shape = ‘ovr’ (one vs. the rest), class_weight = ‘balanced’, and 1000 max iteration: *C* = (1e − 2, 1e − 1, 1e + 0, 1e + 1, 1e + 2, 1e + 3, 1e + 4, 1e + 5, 1e + 6, 1e + 7, 1e + 8, 1e + 9, 1e + 10), gamma = (1e − 9, 1e − 8, 1e − 7, 1e − 6, 1e − 5, 1e − 4, 1e − 3, 1e − 2, 1e − 1, 1e + 0, 1e + 1, 1e + 2, 1e + 3), kernel = (linear, poly, rbf, sigmoid). Its optimized parameters are C = 1.0 and gamma = 0.01 with the RBF kernel. For prediction probabilities, the default outputs are based on Platt scaling^65^ using the *libsvm* library. As Platt scaling is controversial^66^, we also calculate alternative prediction probabilities using normalized *decision_function* scores implemented in *sklearn* based on the optimized parameters for building downstream ensemble models and refer it to SVC2.

(c) RF: We used *RandomForestClassifier* from the *sklearn* library in Python. The following parameter values were used for a grid search of 48 combinations with min_samples_leaf = 2 and the saga solver: n_estimators = (200, 500, 1000); max_depth = (10, 20, 50, 100); criterion = (gini, entropy); max_features = (sqrt, log2). Its optimized parameters are n_estimators = 1000, max_depth = 20, criterion = ‘gini’, and max_features = ‘sqrt’.

(d) XGB: We used *XGBClassifier* from the *xgboost* library in Python. The XGBoost (XGB) framework of gradient boosting trees was the best performing classifier in our previous works^67,68^ as well as in previous studies^69^. The hyperparameter optimization was performed by a grid search of 1620 combinations of the following parameter values: n_estimators = (500, 1000); max_depth = (4, 5, 6); learning_rate = (0.01, 0.1, 0.3, 0.5, 1); gamma = (0.0, 5.0, 10.0); reg_lambda = (0.0, 0.5, 1.0); reg_alpha = (0.0, 0.5, 1.0); subsample = (1.0, 0.75). Its optimized parameters are n_estimators = 1000, max_depth = 5, learning rate = 0.01, gamma = 0.0, reg_lambda = 0.0, reg_alpha = 0.0, and subsample = 0.75.

For the 4 best models with the optimal parameters identified by the above strategy, we next performed more comprehensive training and validation using a repeated multi-fold CV strategy to minimize statistical bias and ensure robustness compared to the single 5-fold CV strategy above. We performed 2-fold, 3-fold, 4-fold, 5-fold, and 10-fold CV with 30, 20, 15, 12, and 6 repetitions with different random seeds, respectively (using *RepeatedStratifiedKFold* from the *scikit-learn* library in Python), i.e., 60 * 5 = 300 CV experiments in total. We denote this strategy as *RMFCV300*.

Next, we built four ensemble models using the four optimized models along with SVC2, as base models, $B=\left\{{\rm{LR}}^{* },{\rm{SVC}}^{* },{\rm{SVC}2}^{* },{\rm{RF}}^{* },{\rm{XGB}}^{* }\right\}$. The rationale for building ensemble models is that ensemble learning has demonstrated success in improving performances over single models in reducing variance or bias^70–72^. From predicted probabilities, *P*_*b*_, of the five base models mapping from each sample, ${s}_{i},{i}=\left\{\mathrm{1,2},\ldots ,n\right\}$ to each class or label, $l\in \left\{1,\,2,\,3,\ldots ,{k}\right\}$, the mean, median, maximum, and minimum for each class were normalized across the four classes as four ensemble models: ${{\rm{MEAN}},\ {\rm{MED}},\ {\rm{MAX}},\ {\rm{and}}\ {\rm{MIN}}}$, respectively, i.e.,

2 $${P}_{\rm{MEAN}}\left(l\right)=\frac{\frac{1}{{||B||}}\sum _{b\in B}{P}_{b}\left(l\right)}{{\sum }_{j}\left\{1/{||B||}{\sum }_{b\in B}{P}_{b}\left(j\right)\right\}}$$

3 $${P}_{\rm{MED}}\left(l\right)=\frac{\mathop{{\rm{med}}}\limits_{b\in B}\left({P}_{b}\left(l\right)\right)}{{\sum }_{j}\mathop{{\rm{med}}}\limits_{b\in B}\left({P}_{b}\left(j\right)\right)}$$

4 $${P}_{\rm{MAX}}\left(l\right)=\frac{\mathop{\max }\limits_{b\in B}\left({P}_{b}\left(l\right)\right)}{{\sum }_{j}\mathop{\max }\limits_{b\in B}\left({P}_{b}\left(j\right)\right)}$$

5 $${P}_{\rm{MIN}}\left(l\right)=\mathop{\min }\limits_{b\in B}\frac{{P}_{b}\left(l\right)}{{\sum }_{j}\mathop{\min }\limits_{b\in B}\left({P}_{b}\left(j\right)\right)}\,$$

Our summary statistics-based ensemble models are a naïve variant of stacked generalization^73^ without additional training. This yielded a nine-classifier system of five optimized base and four ensemble classifiers. We obtained consensus predictions among those nine classifiers as a meta-classifier or a consensus-by-voting system to reduce or average out any bias from a single classifier and improve robustness. The resulting algorithm was designated as *StrokeClassifier*:

6 $$\begin{array}{c}{{StrokeClassifier}}=\Theta =\mathop{\max }\limits_{l}\left(\sum _{\psi }\delta \left(\mathop{\max }\limits_{j}{P}_{\psi }\left(j\right),l\right)\right)\\ \psi \in \left\{{\rm{LR}}^{* },{\rm{SVC}}^{* },{\rm{SVC}2}^{* },{\rm{RF}}^{* },{\rm{XGB}}^{* },{\rm{MEAN},{MED},{MAX},{MIN}}\right\}\\ \delta \left(x,y\right)=\left\{\begin{array}{c}1,\,x=y\\ 0,\,x\,\ne \,y\end{array}\right.\end{array}$$

We additionally analyzed *StrokeClassifier* by (1) training on the YNHH dataset and testing on the MGH and MIMIC datasets and (2) training on the MGH dataset and testing on the YNHH and MIMIC datasets for a five-way cross-hospital validation in total. For the purpose of comparison, we also tested several ensemble models of stacked generalization with the four optimized base models, ${\rm{LR}}^{* },{\rm{SVC}}^{* },{\rm{RF}}^{* },{\rm{XGB}}^{* }$, for the feature group of combn1d.age.sex.v1 (${{\boldsymbol{\Lambda }}}_{{\boldsymbol{1}}}$). We took 11 different combinations of the 4 optimized models as level-0 or base models and each of LR and SVC as the level-1 or meta model. We performed 5-fold CV with seed = 1701 for this purpose.

For model performance evaluation, we used the following 7 performance metrics based on weighted averages for one-vs-rest classification: AUCROC, area under the precision-recall curve (AUPRC or average precision), accuracy (i.e., weighted recall), balanced accuracy (i.e., macro recall or the arithmetic mean of sensitivity and specificity), precision, F1, and Cohen’s kappa. As for the qualitative interpretation of Cohen’s kappa values, we follow the scheme by Landis and Koch^74^: kappa < 0 as no agreement, 0–0.20 as slight, 0.21–0.40 as fair, 0.41–0.60 as moderate, 0.61–0.80 as substantial, and 0.81–1 as almost perfect agreement.

For model interpretation and feature importance, we performed the game-theoretic Shapley value-based SHAP (SHapley Additive exPlanations) analysis using the *shap* package in Python^75,76^, as in our previous works^67,68^. We used TreeSHAP for RF and XGB and KernelSHAP for LR and SVC with a k-means background with k = 100 for computational efficiency. As an alternative approach to ascertain feature importance, we performed classifier-agnostic Kolmogorov–Smirnov tests and Student’s *t*-tests for one-vs-rest comparisons for each class and each feature.

We performed exploratory analyses to evaluate etiologic predictions by *StrokeClassifier* for cryptogenic strokes adjudicated by vascular neurologists. We examined various certainty heuristics defined computationally by thresholds of diagnostic confidence. These diagnostic confidence thresholds were designated by the number of consensus supports provided by the nine individual classifiers in the ensemble model for each non-cryptogenic stroke etiology. As a proof of concept, we applied the threshold of the first quartile of frequencies of support for each etiology from the external validation of the MIMIC-III cohort to predict the etiologies of cryptogenic patients (788 in total) and evaluated the distribution of predicted etiologies. Those predictions with the consensus frequencies less than the thresholds were deemed persistently cryptogenic. We also examined etiology distributions yielded by other quartile thresholds and the means of the support frequencies. Using the first quartile thresholds, we identified a repertoire of EHR signatures associated with each predicted TOAST class for cryptogenic strokes by evaluating feature frequencies from *StrokeClassifier*.

Finally, we performed a longitudinal analysis of *StrokeClassifier* by dividing the combined cohort of YNHH and MGH into a training set of 1,688 discharge summaries from 2015 to 2019 and a test set of 244 discharge summaries from 2020. *StrokeClassifier* was re-trained using the training set along with a stratified 5-fold CV and hyperparameter optimization as above and then longitudinally validated the optimal model using the test set.

All analyses were performed in Python and R using a macOS laptop with 2.6 GHz 6-Core Intel Core i7 and 32GB memory in the case of RF and LR and a high-performance computing cluster with 64 cores and 1GB memory per core in the case of XGB and SVC.

### Reporting summary

Further information on research design is available in the Nature Research Reporting Summary linked to this article.

### Supplementary information

Supplementary Information

Supplementary Tables 1 to 20

Reporting Summary

## Data Availability

The electronic health record data of YNHH and MGH cannot be made available publicly. Sharing this data externally without proper consent could compromise patient privacy and would violate the Institutional Review Board’s approval for the study. MIMIC-III data is publicly available from the PhysioNet repository. We provide full prediction results for the post-processed 499 MIMIC discharge summaries in Supplementary Table 20.

## Code availability

Reasonable requests for the code can be addressed to the corresponding authors.
