# Supplementary material for: StrokeClassifier: ischemic stroke etiology classification by ensemble consensus modeling using electronic health records
Source: NPJ Digit Med. 2024 May 17;7:130. doi: 10.1038/s41746-024-01120-w (PMC11101464; doi:10.1038/s41746-024-01120-w)
Supplement: Supplementary file 1 — Supplementary Information [file 41746_2024_1120_MOESM1_ESM.docx]

**SUPPLEMENTARY INFORMATION**

***StrokeClassifier:*** Ischemic Stroke Etiology Classification by Ensemble Consensus Modeling Using Electronic Health Records

Ho-Joon Lee^1*^, Lee H. Schwamm^2,3^, Lauren Sansing^3^, Hooman Kamel^4^, Adam de Havenon^3^, Ashby C. Turner^2^, Kevin N. Sheth^3^, Smita Krishnaswamy^5^, Cynthia Brandt^6^, Hongyu Zhao^7^, Harlan Krumholz^8^, Richa Sharma^3*†^

^1^ Department of Genetics and Yale Center for Genome Analysis, Yale School of Medicine, New Haven, CT

^2^ Department of Neurology and Comprehensive Stroke Center, Massachusetts General Hospital and Harvard Medical School Boston, MA

^3^ Department of Neurology, Yale School of Medicine, New Haven, CT

^4^ Department of Neurology, Weill Cornell Medicine, New York City, NY

^5^ Departments of Genetics and Computer Science, Yale School of Medicine, New Haven, CT

^6^ Department of Biomedical Informatics and Data Science, Yale School of Medicine, New Haven, CT

^7^ Departments of Biostatistics, Yale School of Public Health, New Haven, CT

^8^ Department of Internal Medicine, Yale School of Medicine, New Haven, CT

**†Lead contact:**

Richa Sharma, M.D., M.P.H.

15 York St., LLCI 1003C

New Haven, CT 06511

Tel: +1-203-737-1057

Email: [Richa.Sharma@yale.edu](mailto:Richa.Sharma@yale.edu)

***Correspondence:** [Richa.Sharma@yale.edu](mailto:Richa.Sharma@yale.edu), [ho-joon.lee@yale.edu](mailto:ho-joon.lee@yale.edu)

**Supplementary Notes**

*A. Stroke sources, targeted therapy, and stroke prevention benefits of non-cryptogenic stroke TOAST classification*

| **TOAST CLASSIFICATION** | **SPECIFIC STROKE SOURCE** | **TARGETED THERAPY** | **STROKE PREVENTION BENEFIT** |
| --- | --- | --- | --- |
| **Large artery atherosclerosis (1)**  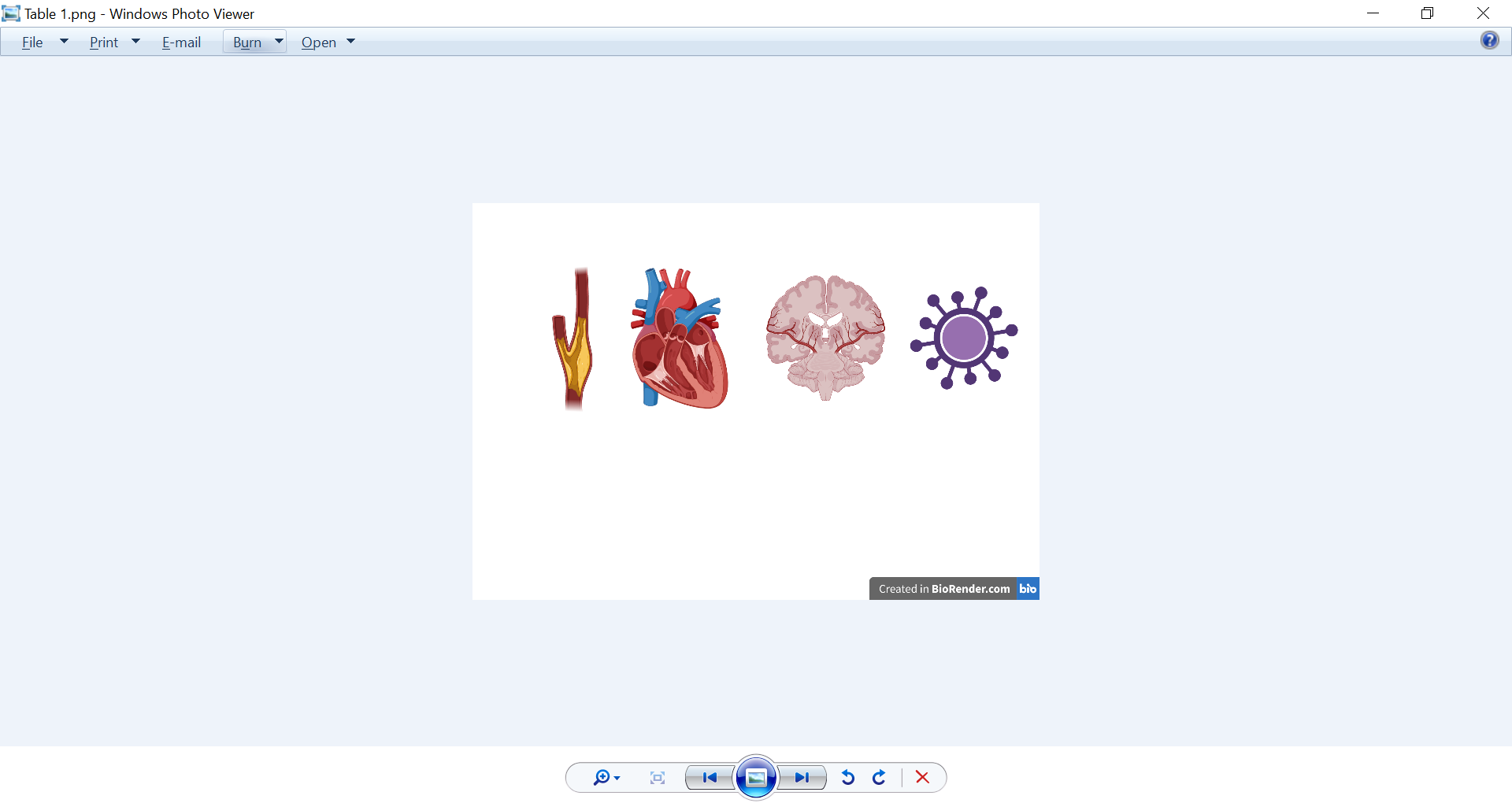 | 1. Symptomatic severely stenotic internal carotid artery 2. Intracranial atherosclerosis | 1. Carotid endarterectomy 2. Dual antiplatelet for 3 months | 1. Absolute risk reduction of ipsilateral stroke in 2 years: **17%**^1^ 2. HR of 30-day stroke or death ticagrelor + aspirin in stroke patients with ipsilateral intracranial stenosis: **0.66** (95% C.I. 0.47-0.93)^2^ |
| **Cardioembolism (2)**  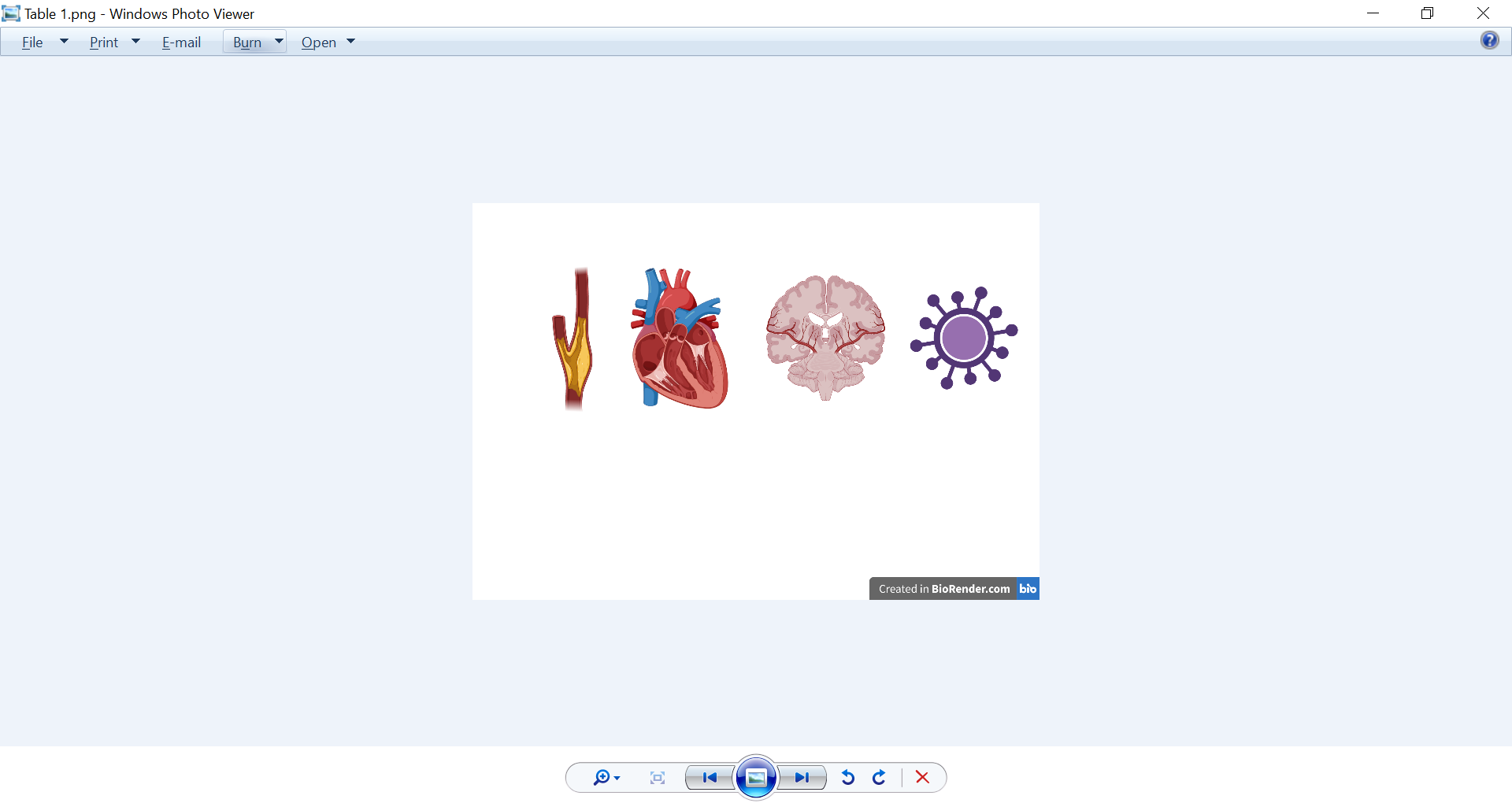 | 1. Atrial fibrillation 2. Endocarditis 3. Patent foramen ovale | 1. Anticoagulation 2. Antibiotics 3. Closure | 1. OR of stroke: **0.56** (95% C.I. 0.41-0.75)^3^ 2. Absolute risk reduction of stroke: **65%**^4^ 3. HR recurrent stroke: **0.23** (95% C.I. 0.09-0.62)^5^ |
| **Small vessel disease (3)**  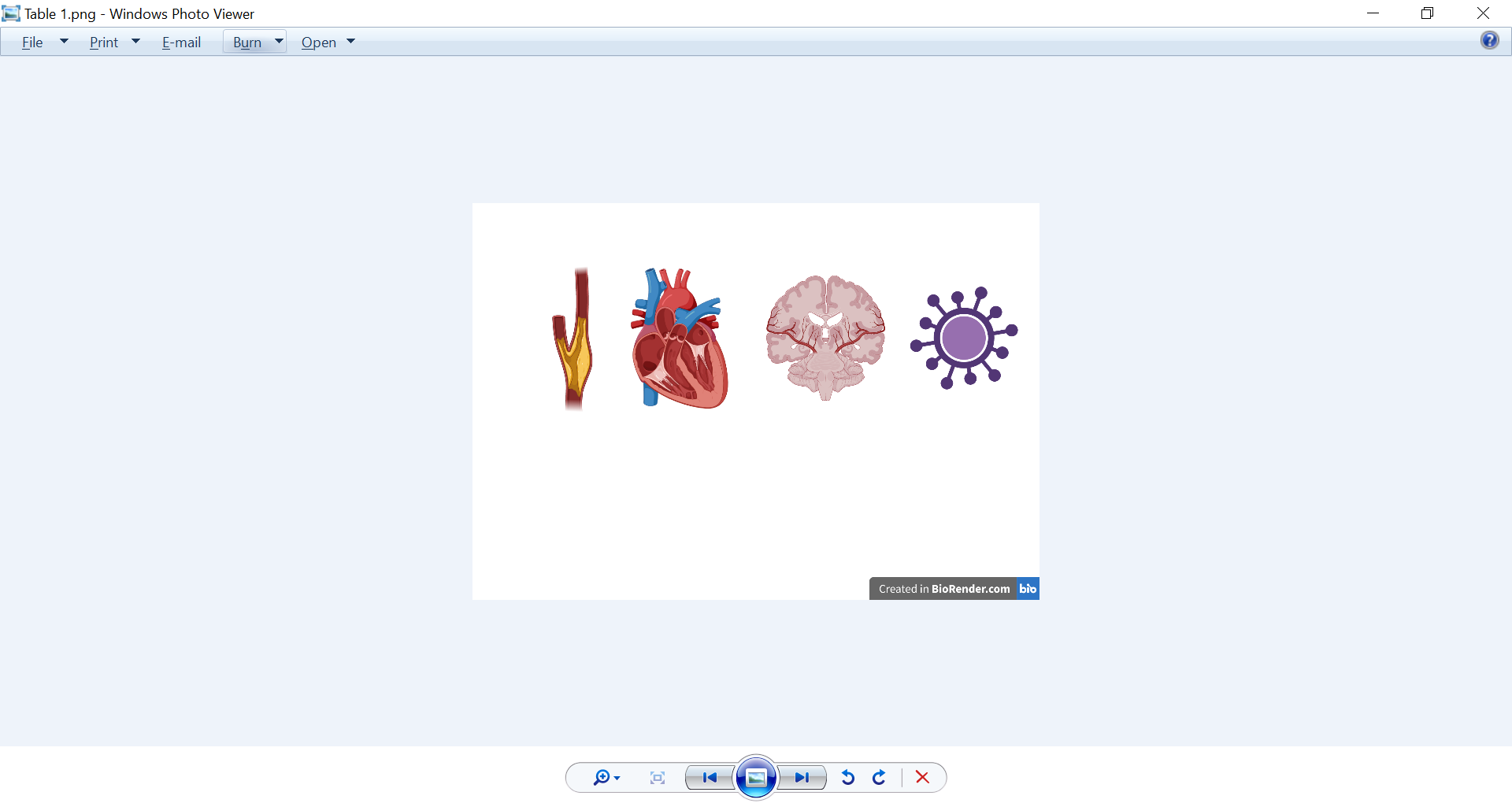 | 1. Small vessel disease | 1. Dual antiplatelet therapy | 1. HR of recurrent stroke with aspirin + clopidogrel: **0.68** (95% C.I. 0.57-0.81)^6^ |
| **Stroke of other determined etiology (4)**  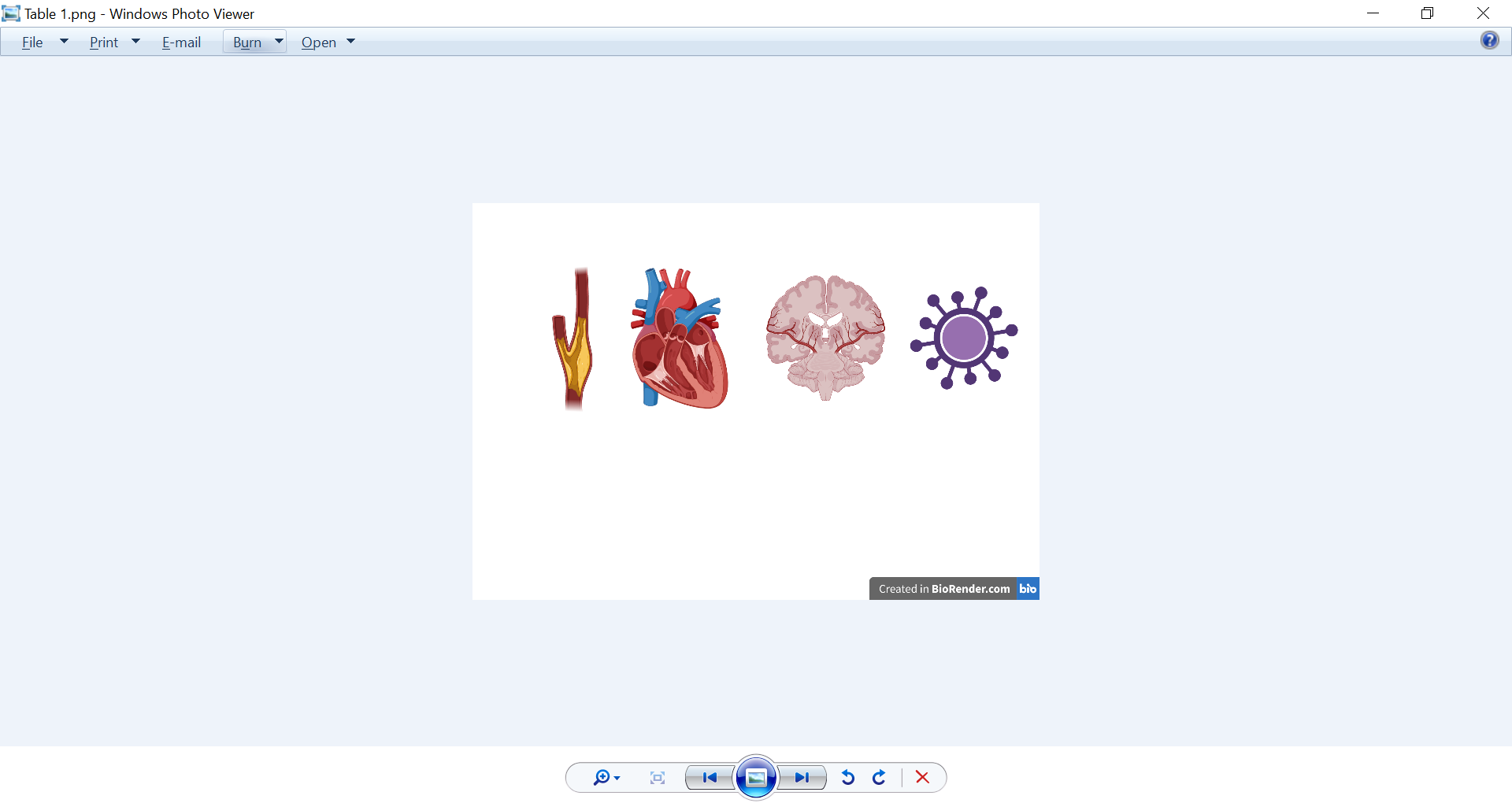 | 1. Hypercoagulability of malignancy | 1. Anticoagulation | 1. HR 1-year survival after stroke with correction of hypercoagulability with anticoagulation among cancer patients: **0.26** (95% C.I. 0.10-0.68)^7^ |

1. [Beneficial Effect of Carotid Endarterectomy in Symptomatic Patients with High-Grade Carotid Stenosis | NEJM](https://www.nejm.org/doi/full/10.1056/nejm199108153250701)
2. [Ticagrelor Added to Aspirin in Acute Nonsevere Ischemic Stroke or Transient Ischemic Attack of Atherosclerotic Origin | Stroke (ahajournals.org)](https://www.ahajournals.org/doi/10.1161/STROKEAHA.120.032239)
3. [Efficacy and Safety of Oral Anticoagulants Versus Aspirin for Patients With Atrial Fibrillation - PMC (nih.gov)](https://www.ncbi.nlm.nih.gov/pmc/articles/PMC4602973/)
4. [The relationship between the initiation of antimicrobial therapy and the incidence of stroke in infective endocarditis: An analysis from the ICE Prospective Cohort Study (ICE-PCS) - ScienceDirect](https://www.sciencedirect.com/science/article/pii/S0002870307006060?via%3Dihub)
5. [Patent Foramen Ovale Closure or Antiplatelet Therapy for Cryptogenic Stroke | NEJM](https://www.nejm.org/doi/full/10.1056/NEJMoa1707404)
6. [Clopidogrel with Aspirin in Acute Minor Stroke or Transient Ischemic Attack | NEJM](https://www.nejm.org/doi/full/10.1056/nejmoa1215340)
7. [The OASIS-CANCER study](https://doi.org/10.5853/jos.2016.00570)

*B. Other models of stacked generalization*

With the 4 optimized base models for the discretized full feature group, *combn1d.age.sex.v1* ($\boldsymbol{\Lambda}_{\boldsymbol{1}}$), we tested several ensemble models of stacked generalization. We investigated 11 different combinations of those 4 optimized models, ${LR}^{*},{SVC}^{*},{RF}^{*},{XGB}^{*}$, as level-0 or base models and each of LR and SVC as the level-1 or meta model. We performed 5-fold CV with seed = 1701. As shown in the box-whisker plots of 5-fold CV below, there were no significant performance differences compared to the optimized base models, except for ${RF}^{*}$ (the prefix, ST|, for stacked models).

(1) LR as a level-1 or meta model


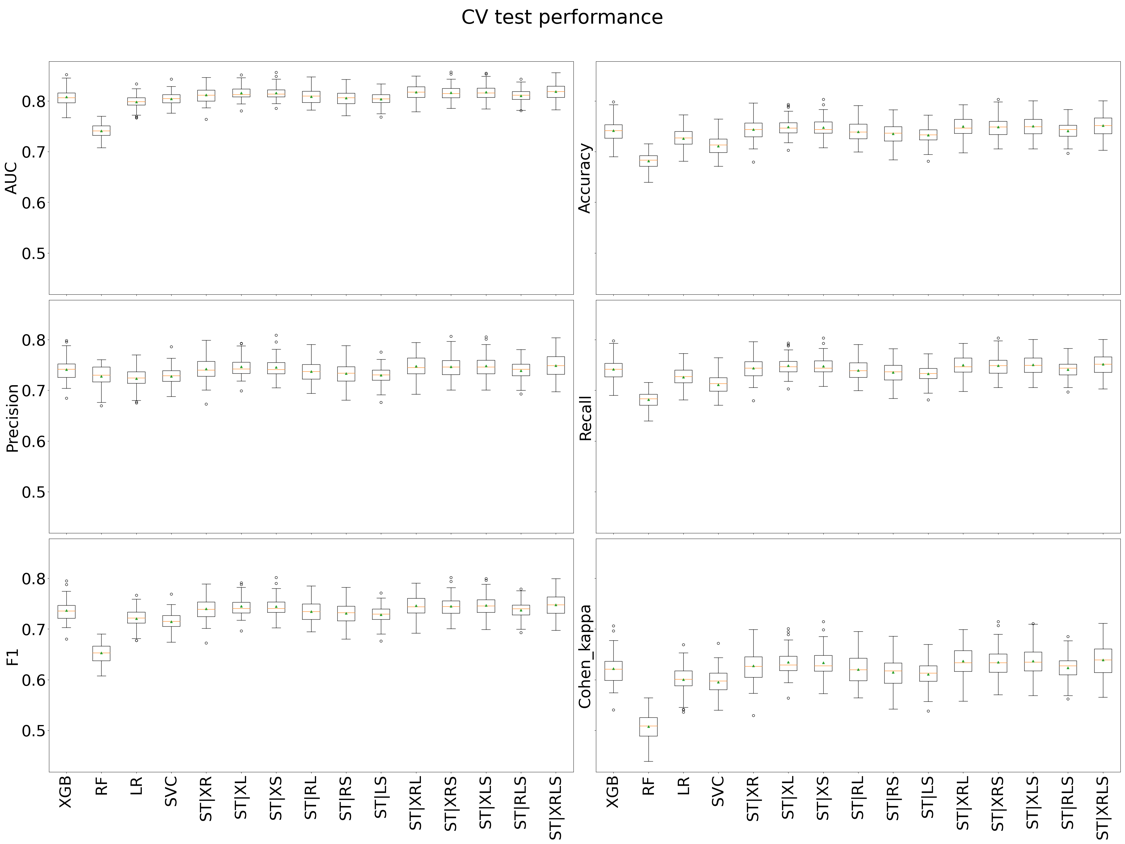


(2) SVC as a level-1 or meta model


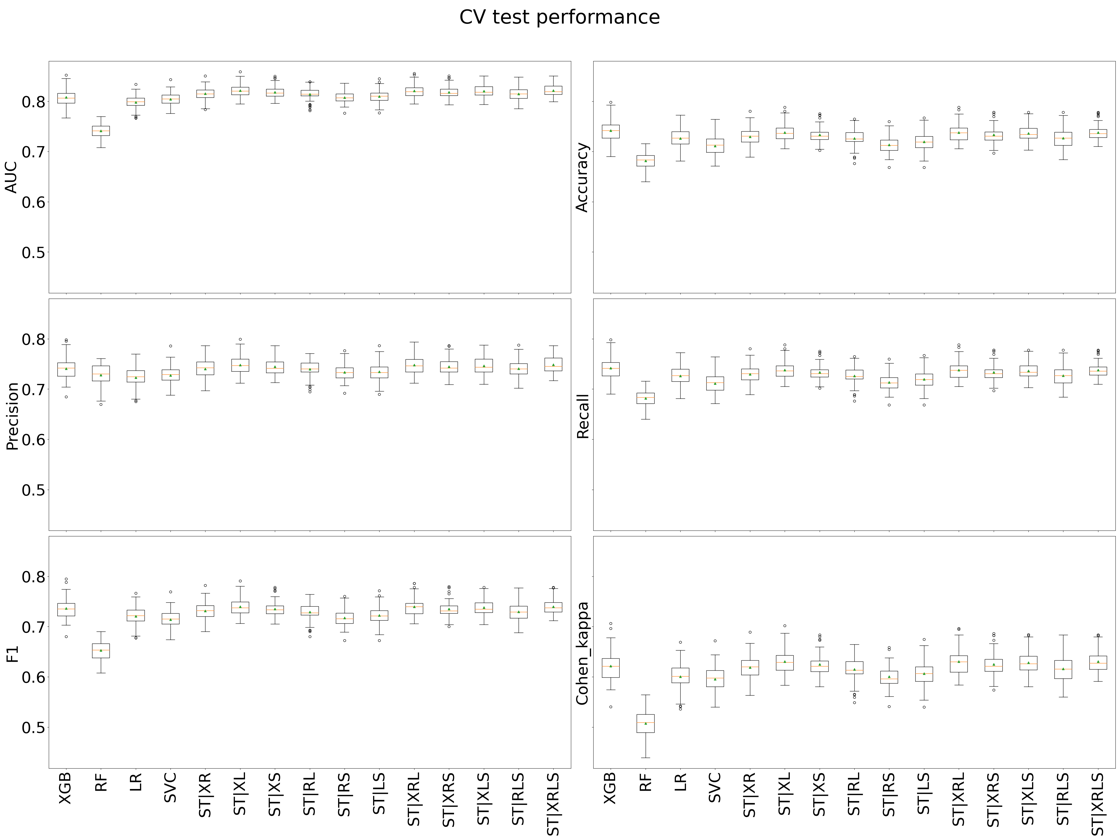


**Supplementary Discussion**

Compared to previous studies on stroke etiology classification, *StrokeClassifier* performed well with respect to a variety of metrics. While Garg et al. reported Cohen’s kappa values of 0.25 using radiology reports alone and 0.57 using combined data (Garg et al., 2019), we achieved highest cross-validated kappa values of 0.400±0.029 using radiology features alone by XGB* and 0.632±0.017 using **Χ(Λ_1_)** by LR* (**Supplementary Table 4**). We note that Cohen’s kappa is considered to be a controversial statistic to quantify agreement between two raters and compare different studies with different categories or classes (Delgado and Tibau, 2019). On the other hand, compared to the c-statistic (i.e., AUCROC) of 0.85 reported by Kamel et al. for a binary classifier (Kamel et al., 2020), the mean c-statistics achieved by the 9 base models used to build *StrokeClassifier* were higher (0.887-0.912; **Table 2b**). We also achieved higher c-statistics for each individual stroke subtype than by Turner et al. (Turner et al., 2022) and comparable c-statistics to Wang et al. (Wang et al., 2022) (**Figure 4a**). More importantly, *StrokeClassifier* outperformed both in terms of predicting stroke etiologies that were in closest agreement with etiologies diagnosed by board-certified vascular neurologists upon review of the entire stroke hospitalization medical record.

Furthermore, in comparison to machine/deep learning classifiers in other diseases (Gui et al., 2022; Shung et al., 2021; Zhan et al., 2021), our current study achieved better classification performances with neither big data nor deep learning, demonstrating robust generalizability. A deep learning study on classification of low vision achieved AUCROC of 82% and AUPRC of 79% (Gui et al., 2022), worse than our RMFCV300 performance with TOAST 2 of similar prevalence of about 40% (AUROC = 90.9±1.2%, AUPRC = 87.9±1.7%; **Supplementary Table 10**). The authors used >5,500 EHRs from a single-center cohort and a different NLP tool to extract CUIs. Another study on prediction of red blood cell transfusion needs for acute gastrointestinal bleeding by a recurrent neural network model of long short-term memory with 2,032 training samples and 62 features showed AUCROC of 81% for internal validation and 65% for external validation (Shung et al., 2021). Machine learning models extracting cardiovascular disease from text performed similarly with significantly more data (Zhan et al., 2021).

There was variability in the predictive capacity of *StrokeClassifier* for each TOAST class. For instance, the model’s accuracy or F1 for predicting cardioembolism (TOAST 2) was lowest at 83% with a high false positive rate of 10% (**Table 3**). The largest contributor to the cardioembolic etiology prediction was AF (**Figure 5**). However, not all ischemic strokes among patients with AF are due to cardioembolism. Patients with AF share risk factors for large artery atherosclerosis and small vessel disease. Further model fine-tuning is necessary to learn the roles of other features and stroke etiology in the context of AF. *StrokeClassifier*’s predictive capacity for stroke etiology also varied by age and sex subgroups. The performance for predicting the large artery atherosclerosis etiology (TOAST 1) per the metrics of the F1 score and balanced accuracy was lower among females, especially those 65 years and older (**Table 4**). The F1 score and balanced accuracy were lower for rare causes of stroke (TOAST 4) among older patients, particularly among older males (**Table 4**). It is unclear what is driving these differences, but we hypothesize that these patterns are reflective of the real-world prevalence of these etiologies within each subgroup (**Supplementary Table 11**) (McKnight et al., 2002).

**Supplementary References**

Delgado, R., and Tibau, X.A. (2019). Why Cohen's Kappa should be avoided as performance measure in classification. PloS one *14*, e0222916.

Garg, R., Oh, E., Naidech, A., Kording, K., and Prabhakaran, S. (2019). Automating Ischemic Stroke Subtype Classification Using Machine Learning and Natural Language Processing. J Stroke Cerebrovasc Dis *28*, 2045-2051.

Gui, H., Tseng, B., Hu, W., and Wang, S.Y. (2022). Looking for low vision: Predicting visual prognosis by fusing structured and free-text data from electronic health records. Int J Med Inform *159*, 104678.

Kamel, H., Navi, B.B., Parikh, N.S., Merkler, A.E., Okin, P.M., Devereux, R.B., Weinsaft, J.W., Kim, J., Cheung, J.W., Kim, L.K.*, et al.* (2020). Machine Learning Prediction of Stroke Mechanism in Embolic Strokes of Undetermined Source. Stroke *51*, e203-e210.

McKnight, L.K., Wilcox, A., and Hripcsak, G. (2002). The effect of sample size and disease prevalence on supervised machine learning of narrative data. Proc AMIA Symp, 519-522.

Shung, D., Huang, J., Castro, E., Tay, J.K., Simonov, M., Laine, L., Batra, R., and Krishnaswamy, S. (2021). Neural network predicts need for red blood cell transfusion for patients with acute gastrointestinal bleeding admitted to the intensive care unit. Scientific reports *11*, 8827.

Turner, A., Spurlock, W., Harrington, L., Khurshid, S., Al-Alusi, M., Reeder, C., Batra, P., Philippakis, A., Lubitz, S., and Anderson, C. (2022). A Machine Learning Approach to Automate Ischemic Stroke Subtyping (N2.001). Neurology *98*, 3538.

Wang, J., Gong, X., Chen, H., Zhong, W., Chen, Y., Zhou, Y., Zhang, W., He, Y., and Lou, M. (2022). Causative Classification of Ischemic Stroke by the Machine Learning Algorithm Random Forests. Frontiers in Aging Neuroscience *14*.

Zhan, X., Humbert-Droz, M., Mukherjee, P., and Gevaert, O. (2021). Structuring clinical text with AI: Old versus new natural language processing techniques evaluated on eight common cardiovascular diseases. Patterns (N Y) *2*, 100289.

**Supplementary Figures**


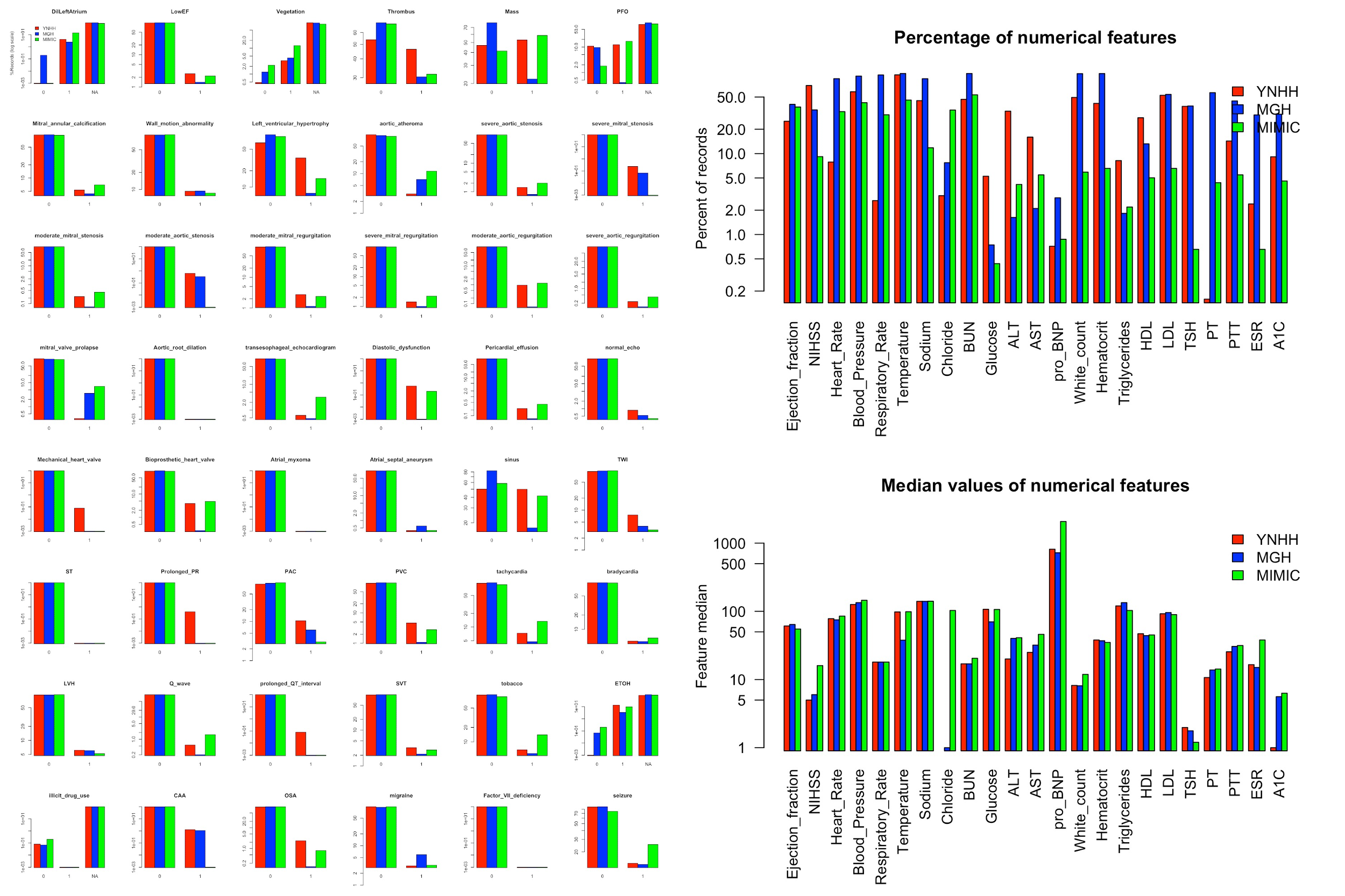


**Supplementary Figure 1. Exploratory data analyses of categorical and numerical features of the 3 cohorts.**


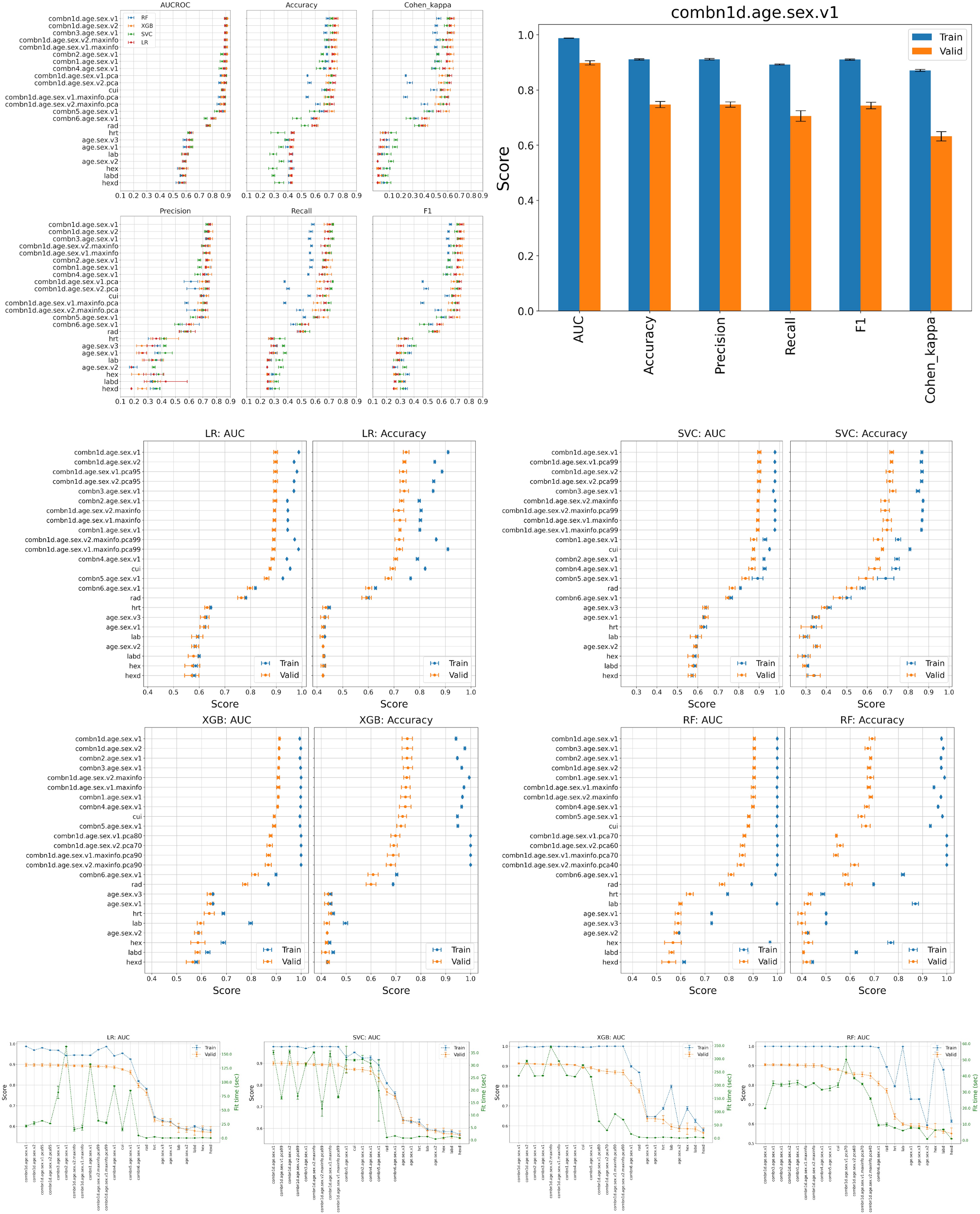


**Supplementary Figure 2. Performance comparisons among all feature groups and the 4 ML algorithms and between the training and validation sets of 5-fold cross validation.** The bar plot is from LR*. The error bars represent mean ± SD of 5-fold CV.


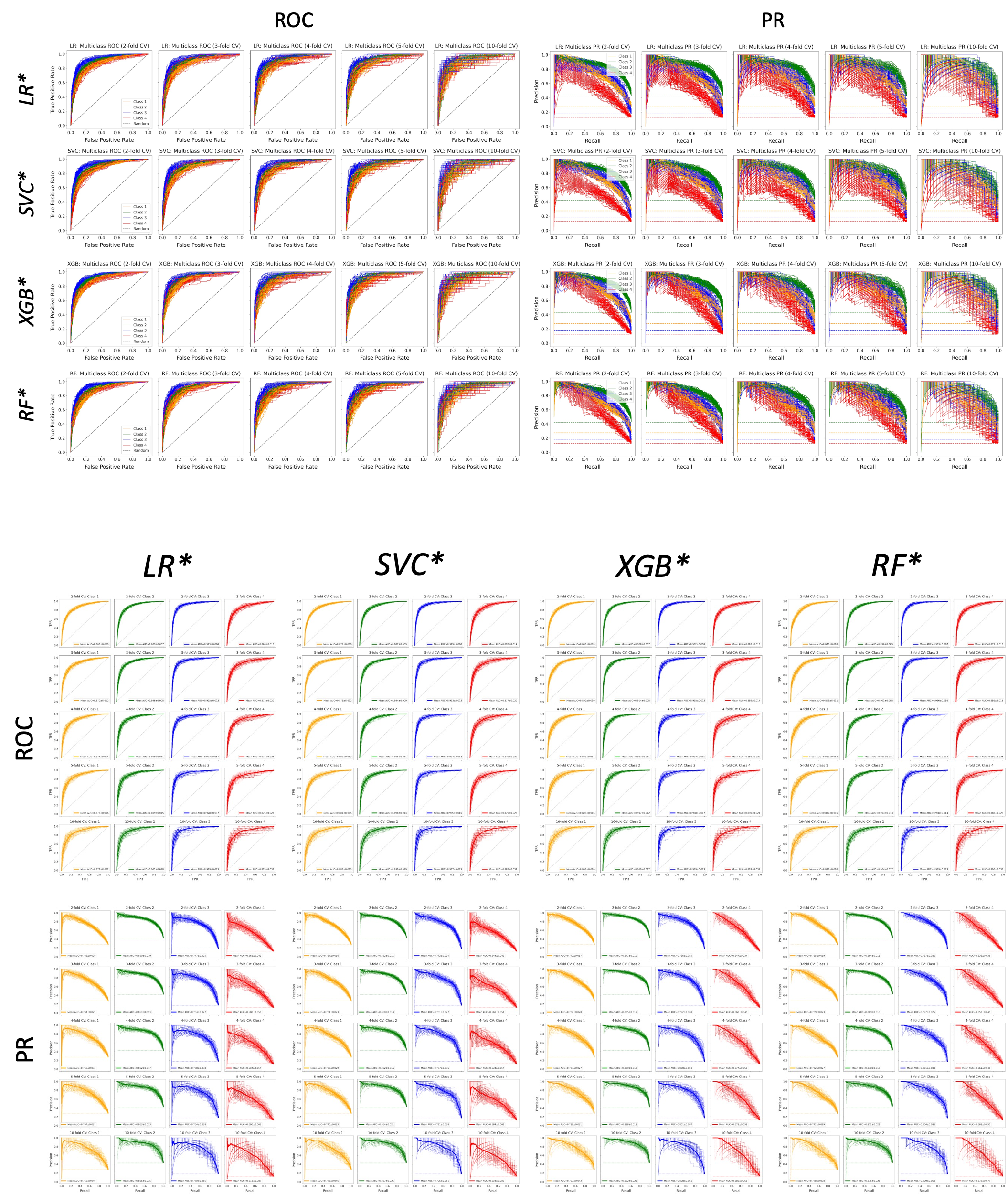


**Supplementary Figure 3. RMFCV300 performances in terms of AUCROC and AUPRC for each optimized model with combn1d.age.sex.v1 (Λ_1_).**


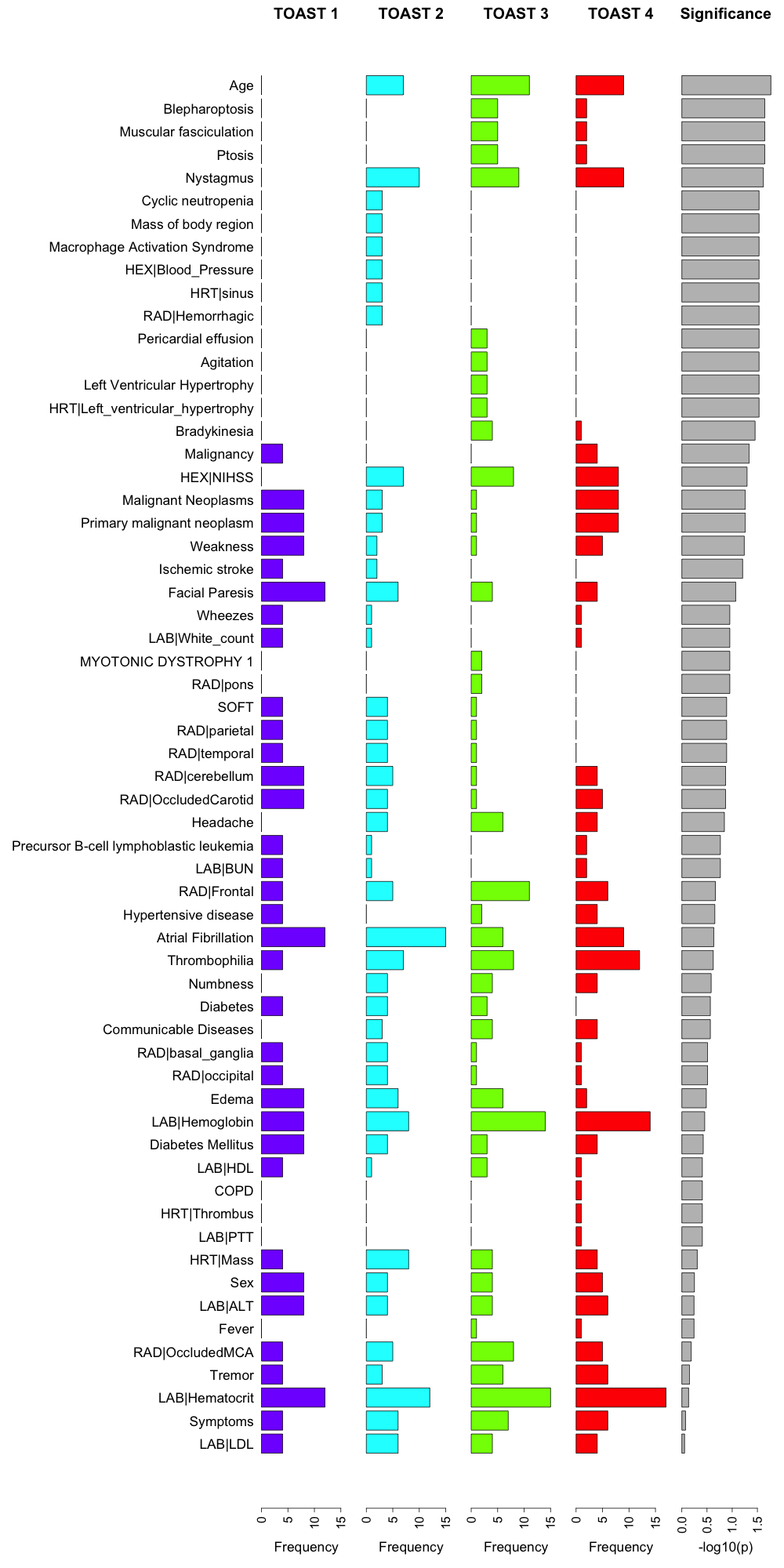


**Supplementary Figure 4. Frequency distributions of the top 10 features contributing to the top 5 PCs by SHAP analysis of the 4 PCA-based optimized models.**


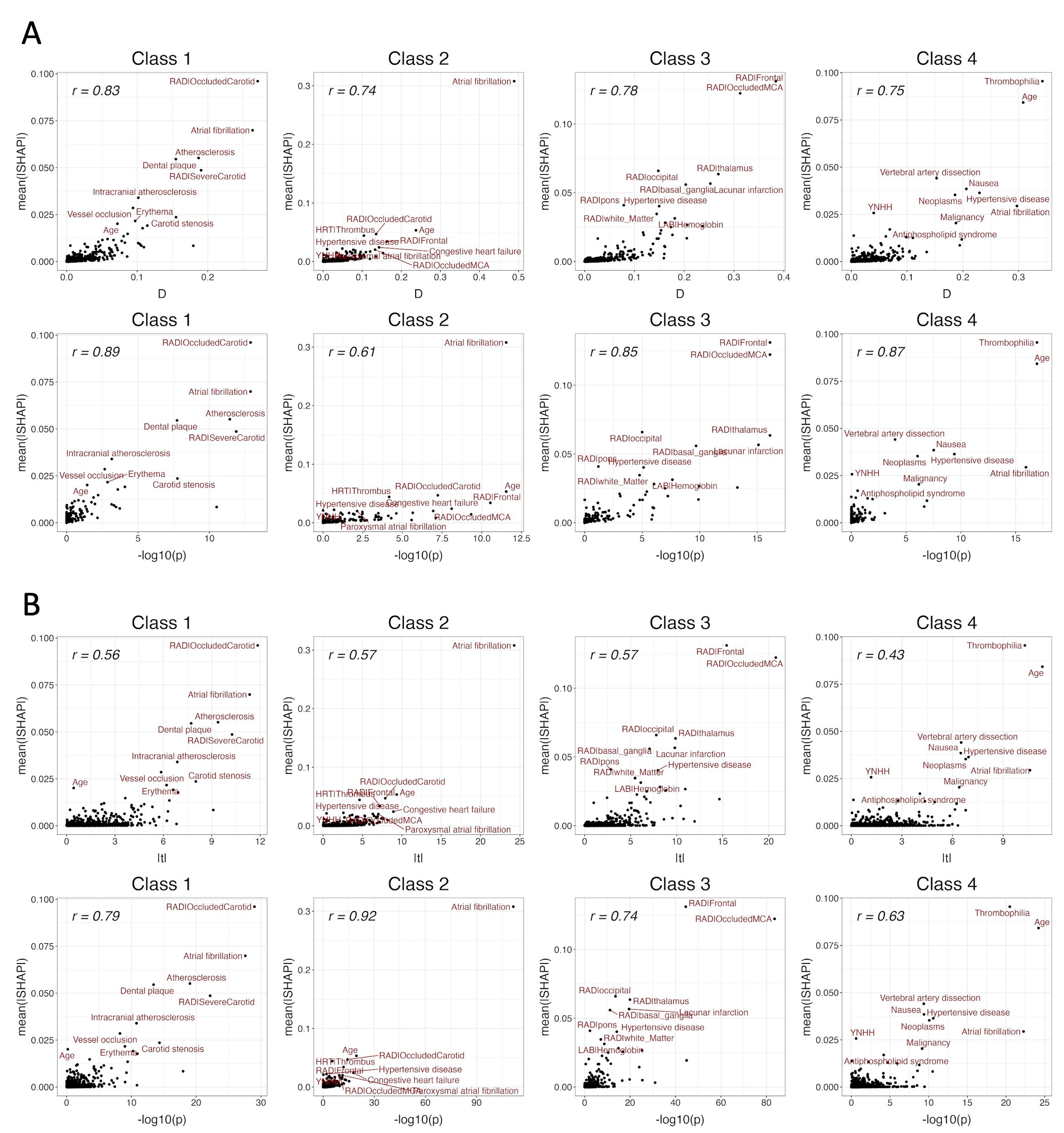


**Supplementary Figure 5. Correlation between SHAP analysis and statistical tests.** (A) Feature correlations between mean(|SHAP|) averaged over the 4 optimized models for each class and the statistic, D (top row), and p-value (bottom row) by Kolmogorov-Smirnov tests for each class vs. the rest. The top 10 features are shown in dark red and Pearson correlation coefficients, *r*, on the top left. (B) Similar analyses to (A) by Student’s t-tests.
