# Supplementary material for: StrokeClassifier: ischemic stroke etiology classification by ensemble consensus modeling using electronic health records
Source: NPJ Digit Med. 2024 May 17;7:130. doi: 10.1038/s41746-024-01120-w (PMC11101464; doi:10.1038/s41746-024-01120-w)
Supplement: Supplementary file 3 — Reporting Summary [file 41746_2024_1120_MOESM3_ESM.pdf]

nature portfolio

Corresponding author(s): Richa Sharma and Ho-Joon Lee

Last updated by author(s): Apr 3, 2024

Reporting Summary

Nature Portfolio wishes to improve the reproducibility of the work that we publish. This form provides structure for consistency and transparency in reporting. For further information on Nature Portfolio policies, see our Editorial Policies and the Editorial Policy Checklist.

Statistics

For all statistical analyses, confirm that the following items are present in the figure legend, table legend, main text, or Methods section.

n/a	Confirmed	
☐	☒	The exact sample size (*n*) for each experimental group/condition, given as a discrete number and unit of measurement
☐	☒	A statement on whether measurements were taken from distinct samples or whether the same sample was measured repeatedly
☐	☒	The statistical test(s) used AND whether they are one- or two-sided<br>*Only common tests should be described solely by name; describe more complex techniques in the Methods section.*
☐	☒	A description of all covariates tested
☐	☒	A description of any assumptions or corrections, such as tests of normality and adjustment for multiple comparisons
☐	☒	A full description of the statistical parameters including central tendency (e.g. means) or other basic estimates (e.g. regression coefficient) AND variation (e.g. standard deviation) or associated estimates of uncertainty (e.g. confidence intervals)
☐	☒	For null hypothesis testing, the test statistic (e.g. *F*, *t*, *r*) with confidence intervals, effect sizes, degrees of freedom and *P* value noted<br>*Give P values as exact values whenever suitable.*
☒	☐	For Bayesian analysis, information on the choice of priors and Markov chain Monte Carlo settings
☒	☐	For hierarchical and complex designs, identification of the appropriate level for tests and full reporting of outcomes
☒	☐	Estimates of effect sizes (e.g. Cohen's *d*, Pearson's *r*), indicating how they were calculated

Our web collection on statistics for biologists contains articles on many of the points above.

Software and code

Policy information about availability of computer code

Data collection	No software used.
Data analysis	R 4.0, Python 3.7, MetaMap, and customized code used.

For manuscripts utilizing custom algorithms or software that are central to the research but not yet described in published literature, software must be made available to editors and reviewers. We strongly encourage code deposition in a community repository (e.g. GitHub). See the Nature Portfolio guidelines for submitting code & software for further information.

Data

Policy information about availability of data

All manuscripts must include a data availability statement. This statement should provide the following information, where applicable:

- Accession codes, unique identifiers, or web links for publicly available datasets
- A description of any restrictions on data availability
- For clinical datasets or third party data, please ensure that the statement adheres to our policy

The patient-level electronic health record data of YNHH and MGH patients analyzed in this observational study cannot be made available publicly. Sharing this data externally without proper consent could compromise patient privacy and would violate the Institutional Review Board approval for the study. MIMIC-III data is publicly available from the PhysioNet repository. We provide full prediction results for the post-processed 499 MIMIC discharge summaries in Supplementary Table 20.

Research involving human participants, their data, or biological material

Policy information about studies with human participants or human data. See also policy information about sex, gender (identity/presentation), and sexual orientation and race, ethnicity and racism.

Reporting on sex and gender	We included biologic sex in model development since there is known sex-specific variation in stroke subtyping. We also evaluated whether there was heterogeneity in model performance based on biologic sex. We assessed model performances in biologic sex strata of male or female per self-report during the acute ischemic stroke hospitalization. This information was abstracted and recorded in each institution's Get-with-the-guidelines Stroke registry.<br><br>In the combined MGH and Yale cohorts, there were 1448 patients who identified as male (52.4%) and 1314 who identified as female (47.5%).
Reporting on race, ethnicity, or other socially relevant groupings	We intentionally did not include the proxy variable of race as a covariate for model training and testing because our datasets lack measures of the social environment which may be more relevant indicators of stroke etiology than ancestry alone.<br><br>To evaluate whether there was heterogeneity in model performancesbased on race, we assessed model performances in race subgroups defined as white, Black, and Other per self-report during the acute ischemic stroke hospitalization. This information was abstracted and recorded in each institution's Get-with-the-guidelines Stroke registry.<br>The distributions of race in the combined MGH and Yale cohorts were: white 1986 (71.9%); Black 380 (13.8%); Other 396 (14.3%)
Population characteristics	The YNHH cohort was significantly older (median age 71 years [IQR 59-82]) compared with the MGH cohort (median age 69 [IQR 59-79]) (p=0.013). The YNHH cohort was significantly more likely than the MGH cohort to have hyperlipidemia (32.9% versus 11.5%, p=0.001) and coronary artery disease (17.8% versus 4.0%, p=0.003). The YNHH and MGH cohorts had similar distributions of stroke etiologies adjudicated by vascular neurologists: large artery atherosclerosis (19.8% versus 21.0%), cardioembolism (32.9% versus 29.9%), small vessel disease (15.3% versus 10.7%), other determined etiology (8.9% versus 9.6%), and cryptogenic etiology (23.1% versus 28.8%).
Recruitment	Acute ischemic stroke hospitalizations at YNHH and MGH were identified by each institution's Get-with-the-guidelines stroke database. Get-With-The-Guidelines (GWTG)-Stroke database is a quality improvement initiative in which participating hospitals enter clinical and radiographic data of all patients hospitalized with an ischemic stroke diagnosis38. Acute ischemic stroke patients are identified by administrative billing codes (International Classification of Diseases (ICD), 10th Revision). Data abstraction, entry, and adjudication are performed by trained study personnel. There are logic checks and form controls to minimize data entry errors. The database was queried for all ischemic stroke patients >18 years admitted from January 2015 to December 2020 at MGH and YNHH to assemble the ischemic stroke cohort. The EHR platform for both institutions is Epic (Epic Systems Corporation), the most prevalent EHR system in the United States. Stroke hospitalizations from the GWTG databases were linked with corresponding semi-structured discharge summary plain ASCII text files, resulting in a total 1,269 and 1,493 records from YNHH and MGH, respectively.
Ethics oversight	This study was approved by the Institutional Review Boards of Massachusetts General Hospital and Yale-New Haven Hospital.

Note that full information on the approval of the study protocol must also be provided in the manuscript.

Field-specific reporting

Please select the one below that is the best fit for your research. If you are not sure, read the appropriate sections before making your selection.

☒ Life sciences ☐ Behavioural & social sciences ☐ Ecological, evolutionary & environmental sciences

For a reference copy of the document with all sections, see nature.com/documents/nr-reporting-summary-flat.pdf

Life sciences study design

All studies must disclose on these points even when the disclosure is negative.

Sample size	The sample size was determined by the availability of data from patients who met inclusion and exclusion criteria during the timeframe of data collection. No sample-size calculation was performed. The sample size was sufficient based on the previous studies of machine learning.
Data exclusions	A subset of data was excluded if there was processing failure by MetaMap.
Replication	The reproducibility of the findings was verified by using the same random seed.
Randomization	The samples were randomized by a randomization algorithm in Python.
Blinding	The investigators were blinded because the data were deidentified.

Reporting for specific materials, systems and methods

We require information from authors about some types of materials, experimental systems and methods used in many studies. Here, indicate whether each material, system or method listed is relevant to your study. If you are not sure if a list item applies to your research, read the appropriate section before selecting a response.

Materials & experimental systems

n/a	Involved in the study
☒	☐ Antibodies
☒	☐ Eukaryotic cell lines
☒	☐ Palaeontology and archaeology
☒	☐ Animals and other organisms
☒	☐ Clinical data
☒	☐ Dual use research of concern
☒	☐ Plants

Methods

n/a	Involved in the study
☒	☐ ChIP-seq
☒	☐ Flow cytometry
☒	☐ MRI-based neuroimaging

Plants

Seed stocks	*Report on the source of all seed stocks or other plant material used. If applicable, state the seed stock centre and catalogue number. If plant specimens were collected from the field, describe the collection location, date and sampling procedures.*
Novel plant genotypes	*Describe the methods by which all novel plant genotypes were produced. This includes those generated by transgenic approaches, gene editing, chemical/radiation-based mutagenesis and hybridization. For transgenic lines, describe the transformation method, the number of independent lines analyzed and the generation upon which experiments were performed. For gene-edited lines, describe the editor used, the endogenous sequence targeted for editing, the targeting guide RNA sequence (if applicable) and how the editor was applied.*
Authentication	*Describe any authentication procedures for each seed stock used or novel genotype generated. Describe any experiments used to assess the effect of a mutation and, where applicable, how potential secondary effects (e.g. second site T-DNA insertions, mosiacism, off-target gene editing) were examined.*
